# HCMV Variants Expressing ULBP2 Enhance the Function of Human NK Cells via its Receptor NKG2D

**DOI:** 10.1002/eji.202451266

**Published:** 2025-02-11

**Authors:** Greta Meyer, Anna Rebecca Siemes, Jenny F. Kühne, Irina Bevzenko, Viktoria Baszczok, Jana Keil, Kerstin Beushausen, Karen Wagner, Lars Steinbrück, Martin Messerle, Christine S. Falk

**Affiliations:** ^1^ Institute of Transplant Immunology Hannover Medical School Hannover Germany; ^2^ Institute of Virology Hannover Medical School Hannover Germany; ^3^ German Center for Infection Research (DZIF) TTU‐IICH (Infection of the immunocompromised host) Hannover/Braunschweig Germany

**Keywords:** attenuation, HCMV vaccine, NK cells, NKG2D, ULBP2

## Abstract

The immunosuppressed state of transplant patients allows opportunistic pathogens such as human cytomegalovirus (HCMV) to cause severe disease. Therefore, inducing and boosting immunity against HCMV in recipients prior to organ transplantation is highly desirable, and accordingly, the development of an HCMV vaccine has been identified as a clinically relevant priority. Such vaccines need to be highly attenuated while eliciting specific and protective immune responses. We tested the concept of expressing the NKG2D ligand (NKG2D‐L) ULBP2 by HCMV vaccine candidates to achieve NK cell activation, and, thereby viral attenuation. ULBP2 expression was found on HCMV‐infected cells, reflecting the promotor strengths used to drive ULBP2 transgene expression. Moreover, significantly increased shedding of soluble ULBP2 (sULBP2) was detected for these mutants mirroring the surface expression levels. No negative effect of sULBP2 on NK cell function was observed. NK cells efficiently controlled viral spread, which was further increased by additional triggering of the activating receptor NKG2D. Engagement of NKG2D was also confirmed by its downregulation depending on ULBP2 surface density. Finally, expression of ULBP2 significantly enhanced NK cell cytotoxicity, which was independent of KIR‐ligand mismatch as well as the presence of T cells. This NKG2D‐L‐based approach represents a feasible and promising strategy for HCMV vaccine development.

## Graphical abstract

As an approach for HCMV vaccine development, HCMV mutants expressing the NKG2D ligand ULBP2 were generated. Expression of ULBP2 by virus‐infected cells enabled specific interaction with the activating receptor NKG2D resulting in enhanced NK cell degranulation capacity.

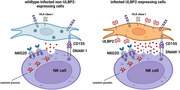

**Abbreviations**
BAC: bacterial artificial chromosome
DNAM‐1: DNAX accessory molecule 1
dpi: day/days postinfection
E:T ratio: effector:target ratio
FMO: fluorescence minus one
HCMV: human cytomegalovirus
HFF: human foreskin fibroblasts
ICAM‐1: intercellular adhesion molecule‐1
MICA/MICB: MHC class I polypeptide‐related sequence A/B
MIEP: major immediate‐early promoter
NK cells: natural killer cells
NKG2A/C/D: NK group 2 member A/C/D
NKG2D‐L: NKG2D ligand
ORF: open reading frame
p.i.: postinfection
PBMC: peripheral blood mononuclear cells
TIGIT: T cell immunoreceptor with immunoglobulin and ITIM
ULBP: UL16‐binding protein
VDA: viral dissemination assay

## 1 Introduction

Transplant patients who received a solid organ or hematopoietic stem cells are at high risk of developing serious, life‐threatening disease, resulting from primary human cytomegalovirus (HCMV) infection or reactivation [1]. HCMV can be transmitted with the transplanted organ or cells, or it can reactivate from latent infection previously established in the recipient. In case of insufficient immune control, HCMV disseminates to various organs and tissues, leading to illnesses such as retinitis, hepatitis, colitis or interstitial pneumonia, and even multiorgan failure and death [1]. Moreover, reactivation events trigger acute and chronic graft rejection, gradually diminishing the function of the grafted organ or leading to graft loss [1, 2].

Inducing or boosting immunity to HCMV would, therefore, be of great benefit for transplant patient groups, for example, patients on waiting lists for kidney transplantation. In support of the vaccination idea, an increased CMV‐specific T‐cell response often correlates with a reduced incidence of reactivation episodes [3, 4]. Development of a protective HCMV vaccine has been a strategic goal since the early 1970s, with approaches ranging from subunit vaccines, over DNA and vectored vaccines to attenuated life vaccines [5]. In kidney and liver transplant recipients, a subunit vaccine led to a reduction in disease burden and duration of viremia, but it did not provide complete protection against infection and disease [6]. Live vaccines are considered to elicit broader and long‐lasting immunity; however, such vaccine candidates need strong attenuation and have to be further evaluated [7]. Until now, none of the investigated HCMV vaccine approaches have been approved for clinical application, especially for transplant patient groups.

Induction of protective immunity against HCMV by vaccination may be particularly challenging because this virus has evolved multiple mechanisms to interfere with immune control (reviewed in [8, 9]). For instance, HCMV encodes several HLA class I immune evasins (US2, US3, US6, US10, and US11), all of which interfere with viral antigen presentation, hence, affecting recognition by specific CD8^+^ T cells [9]. Similarly, HCMV has developed various strategies to prevent recognition and cytolysis by natural killer (NK) cells [10, 11], and of note, other species of CMVs have acquired comparable immune‐evasive properties [12, 13]. In particular, CMVs avoid expression of stress‐ or infection‐induced ligands for activating NK cell receptors on the surface of infected cells [14, 15], especially the ligands ULBP1, ULBP2, MICA, and MICB for the human activating NKG2D receptor [16, 17, 18, 19].

In an attempt to reverse this immune escape, the NKG2D ligands RAE‐1γ and MULT‐1 were expressed by mouse CMV (MCMV) mutants [20, 21]. These variants turned out to be highly attenuated, in a NK cell‐mediated manner, and despite attenuation, they could trigger strong MCMV‐specific CD8^+^ T cell responses. Such properties are highly attractive for vaccine development, and the feasibility of these MCMV variants as vaccine vectors has been corroborated in different mouse models [22, 23]. Although the underlying immunological mechanisms are still under investigation [24], we have started to translate this concept to HCMV and generated an ULBP2‐expressing HCMV mutant [25]. The first evaluation of this HCMV mutant pointed towards favorable vaccine properties, particularly its attenuation via NK cells, however, several aspects needed further investigation and were addressed in this study.

First, it remained unclear whether the very strong ULBP2 expression in fibroblasts infected with the mentioned HCMV ULBP2 variant [25] was necessary for NK cell activation, and second, whether it was associated with shedding of soluble ULBP2, which in turn, could interfere with NKG2D functions. Moreover, this strong ULBP2 expression was shown to further reduce HLA class I surface expression [25], although this mutant lacks the immune evasins US2‐6. Therefore, we generated additional HCMV variants, which expressed ULBP2 under a weaker promoter and differed in the number of HLA class I immune evasins, that is, the US2‐6 region was reconstituted. These novel ULBP2‐expressing HCMV variants allowed us to determine the impact of the ULBP2 expression density on infected fibroblasts on NK cell activation, NKG2D interaction, and control of viral spread in the presence of NK cells.

## 2 Results

### 2.1 HCMV TB40 Variants for Assessing the Role of NKG2D‐Mediated Co‐Stimulation of NK Cells

**FIGURE 1:**
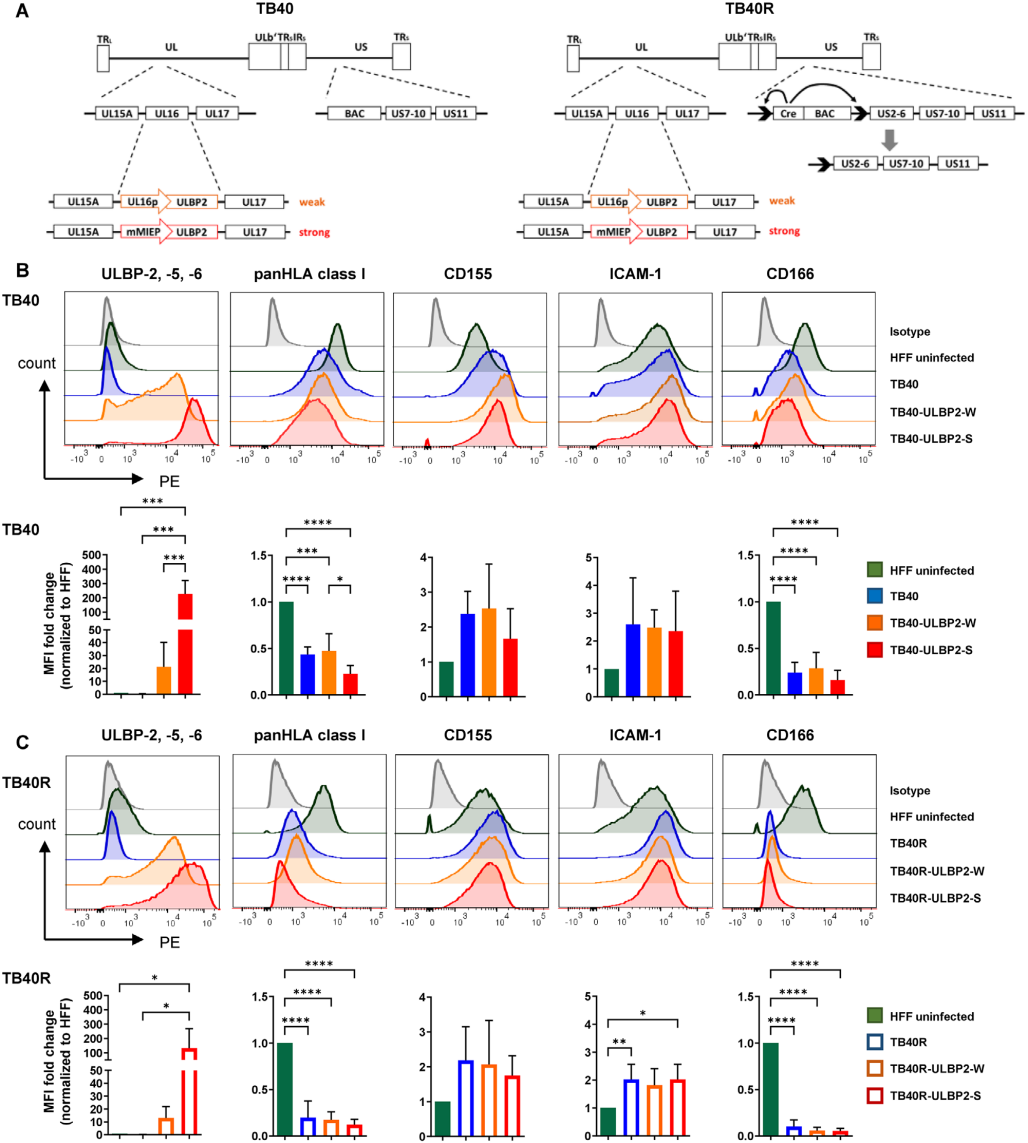
Characterization of NKGD2 ligand‐expressing HCMV variants. (A) Schematic representation of the genome structures of the used virus variants that are based on the BAC‐cloned HCMV strains TB40 (ΔUS2‐US6) [26] and TB40R [27]. In each case, the viral UL16 ORF was replaced by the coding region for the NKG2D‐L ULBP2 that was expressed either under control of the UL16 promotor (termed W variants) or the major immediate‐early promotor (MIEP) (termed S variants). (B, C) Uninfected HFF and cells infected with the different HCMV variants for 4 days were analyzed by flow cytometry for expression of the indicated surface molecules. Median fluorescence intensities (MFI) are represented as overlays and as ΔMFI fold change (MFI—isotype) normalized to MFI of uninfected HFF (mean ± SD, *n* = 3–6). Statistical analysis was performed by one‐way ANOVA with Tukey's multiple comparison test, and statistical significance is indicated as *p < 0.05, **p < 0.01, ***p < 0.001, ****p < 0.0001.

Previous findings in MCMV [20, 21, 22] indicated that viral expression of NKG2D ligands (NKG2D‐L) can lead to co‐stimulation and activation of NK cells, which limit viral spread due to enhanced cytotoxicity and cytokine secretion, hence resulting in attenuation. To test this concept for HCMV, we constructed novel HCMV variants expressing the NKG2D‐L ULBP2, which were derived from the BAC‐cloned HCMV strains TB40 [26] and TB40R [27]. TB40‐ULBP2‐W and TB40‐ULBP2‐S are based on the TB40‐BAC4 [25, 26] and lack the HLA class I immune evasins US2 to US6 (TB40 ΔUS2‐6, Figure 1A), which may alleviate downmodulation of HLA class I molecules. The major‐immediate early promoter (MIEP) used in TB40‐ULBP2‐S is known to provide high ULBP2 transgene expression. In order to generate a variant with lower ULBP2 expression, we constructed TB40‐ULBP2‐W in which the viral UL16 promoter drives the ULBP2 gene. In both variants, the UL16 ORF was deleted (and replaced by the ULBP2 gene), to disrupt the UL16 effect on NKG2D‐L surface expression and prevent interference with ULBP2 expression. Furthermore, two analogous virus variants, termed TB40R‐ULBP2‐S and –W, were constructed based on the TB40R strain [27], which encode the entire set of HLA class I immune evasins US2 to US11 (Figure 1A). In the TB40R‐based virus mutants, the BAC vector was self‐excised from the BAC‐cloned virus genomes upon reconstitution of infectious viruses to avoid potential adverse effects as reported by Murrell et al. [28]. These virus variants grew with identical kinetics compared with the parental virus (Figure S1A), in agreement with the non‐essential role of UL16 and the expectation that ULBP2 does not interfere with HCMV replication.

### 2.2 Gradual Expression of ULBP2 and HLA Class I by Fibroblasts Infected with the Different HCMV Variants

Human foreskin fibroblasts (HFF) infected with the TB40 and TB40R mutants were phenotypically characterized by flow cytometry with respect to the expression of the transgene ULBP2, HLA class I, and other surface molecules (Figure 1B,C). Cells infected with the TB40‐ULBP2‐S and TB40R‐ULBP2‐S mutants expressed more than 100‐fold higher ULBP2 amounts compared with uninfected HFF. Upon infection with the corresponding TB40‐ or TB40R‐ULBP2‐W mutants, ULBP2 levels increased up to 44‐fold, reflecting the differential strength of the two promoters. As expected, cells infected with the parental TB40 or TB40R strains did not show increased ULBP2 expression due to UL16‐mediated retention [29]. The presence of the immune evasins US10 and US11 in the TB40 (ΔUS2‐6) strains was sufficient to downregulate HLA class I surface expression (Figure 1B). Likewise, HLA class I levels were significantly decreased upon infection with the TB40R‐based strains expressing all immune evasins (Figure 1C; Figure S1B). Moreover, there was a tendency towards even lower HLA class I levels following infection with the ULBP2‐S virus variants compared with the ULBP2‐W variants and parental viruses (Figure 1B,C).

Surface levels of the adhesion molecules ICAM‐1 and CD155, the ligand for the activating and inhibitory NK cell receptors DNAM‐1 and TIGIT, were slightly upregulated on all infected cells independently of ULBP2 expression (Figure 1B,C; Figure S1B). Surface expression of CD166, a cell adhesion molecule and CD6 ligand, were significantly downregulated upon infection with viruses of both genetic backgrounds. Due to the deletion of the UL16 ORF in the ULBP2‐expressing mutants, induction of other NKG2D‐L was expected upon infection, and, hence, slightly increased ULBP1 expression was observed in HFF infected with ULBP2‐W and ULBP2‐S variants (Figure S1C,D). As expected, MICA was downregulated on all virus‐infected cells compared with uninfected HFF, since it is not a target of UL16. The other NKG2D‐L ULBP3 and MICB could not be detected on these HFF (data not shown). HLA‐E, the ligand of the heterodimers CD94/NKG2A and CD94/NKG2C, showed generally low expression in uninfected HFF and infection with all variants led to further downregulation, indicating activity of immune evasins within the US2‐US11 region [30].

Here, we could show that ULBP2 expression in infected HFF was dependent on weak or strong promoter activity, respectively, and that, simultaneously, HLA class I downregulation was influenced by the presence of the viral immune evasins US2‐11. Other NKG2D‐L were also modulated as a consequence of the presence or absence of UL16 and these immune evasins.

### 2.3 Soluble ULBP2 Does Not Affect Cytotoxicity and NKG2D Receptor Modulation of NK Cells

**FIGURE 2:**
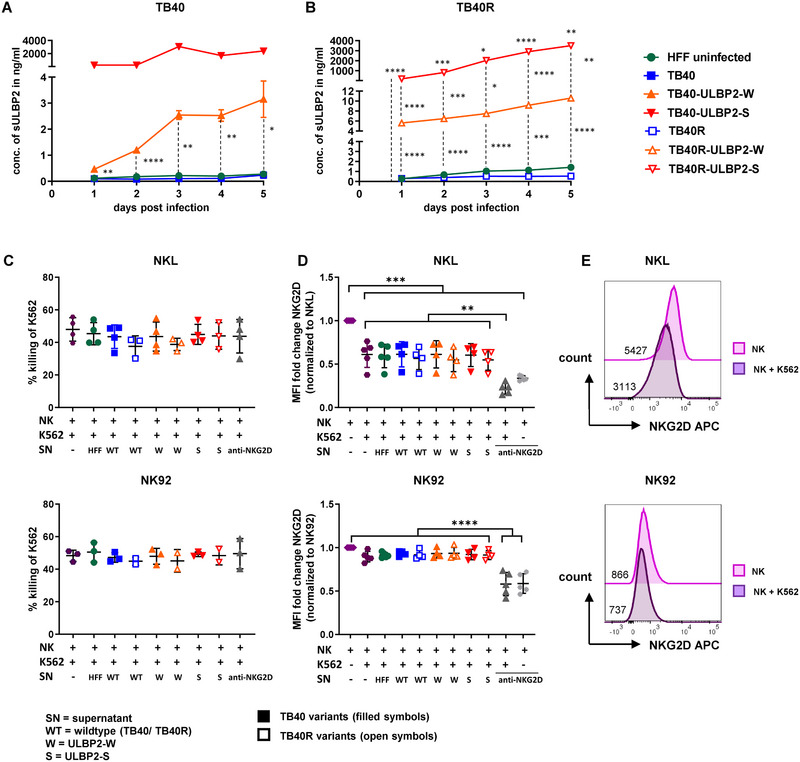
Shedding of soluble ULBP2 by HCMV‐infected HFF. (A, B) The amount of soluble ULBP2 shed in culture supernatants by uninfected HFF or HCMV‐infected cells over 5 days was assessed by ULBP2‐ELISA (*n* = 3 biological triplicates per condition, depicted as mean ± SD; except for TB40‐ULBP2‐S: only unique samples were analyzed per time point). (C) Killing of K562 cells (in %; mean ± SD, *n* = 2–4) upon contact with NKL or NK92 cells and addition of indicated culture supernatants. (D) MFI fold change of NKG2D receptor expression (mean ± SD, *n* = 2–4) upon contact with K562 and culture supernatants normalized to MFI of NKL/NK92 cells kept in the absence of K562 cells and supernatant. As control, an NKG2D antibody (5 µg/mL) was used to block the receptor (grey symbols). (E) Representative overlay of the baseline NKG2D expression of NKL/NK92 cells and upon contact with K562 target cells. Representative gating strategy is shown in Figure S3A. Significance values for (A, B) were determined by two‐way ANOVA with Tukey´s multiple comparison test and Geisser–Greenhouse correction. Significance values for (C, D) were determined by one‐way ANOVA with Tukey's multiple comparison test. *p < 0.05, **p < 0.01, ***p < 0.001, ****p < 0.0001.

ULBP2 belongs to those NKG2D ligands, which can be shed in a cell‐type‐specific manner due to proteolytic cleavage and release into the extracellular environment [31, 32]. Soluble ULBP2 (sULBP2) may interact with the NKG2D receptor on NK cells, leading to its downregulation or masking, potentially interfering with NKG2D binding to target cells. Quantification of sULBP2 in culture supernatants of infected cells by ELISA revealed strong accumulation during 5‐day culture (Figure 2A,B). For both, TB40 and TB40R variants, sULBP2 levels mirrored the ULBP2 cell surface expression, reflecting the strength of the MIEP and UL16 promotor, respectively. Culture supernatants of uninfected HFF or cells infected with the parental strains contained low amounts of sULBP2.

Despite high levels of soluble ULBP2, surface expression remained constantly high, indicating continuous ULBP2 expression as a membrane‐bound molecule (Figure S2A,B). Other soluble NKG2D‐L were either barely detectable or only at low levels in supernatants of infected cells (Figure S2C). In contrast, CD155 was secreted by virus‐infected cells, in line with its upregulation at the cell surface (compare Figure 1B,C).

Next, we examined the potential effects of sULBP2 on NK cell function. The NK cell lines NKL and NK92 were preincubated with sULBP2‐containing culture supernatants and co‐cultured with HLA class I‐deficient CFSE‐labeled K562 target cells (for gating strategy see Figure S3A), known to be highly susceptible to NK cell‐mediated lysis. sULBP2 levels in the co‐culture samples were verified by ULBP2‐ELISA (Figure S3B). Cytotoxicity against K562 cells was not significantly altered in the presence of sULBP2 (Figure S2C), suggesting that the soluble ligands do either not directly interact with NKG2D or do not block the recognition and killing of K562. Similarly, the addition of a NKG2D‐specific antibody did not block K562 killing, probably due to other receptors involved in cytotoxicity. However, the NKG2D receptor levels at the surface of NK92 and NKL cells were not altered in the presence of sULBP2. In NKL cells, contact with K562 target cells resulted in stronger receptor internalization compared with NK92 (Figure 2D,E). As expected, the addition of unlabeled anti‐NKG2D antibodies led to reduced NKG2D MFI fold changes due to blockade of the NKG2D receptor.

To test whether sULBP2 can directly bind to NKG2D, NKL cells were preincubated with sULBP2‐containing supernatants, and subsequent binding of an ULBP2‐Fc fusion protein was used as a readout for sULBP2 interaction with NKG2D. As a second receptor/ligand pair, CD155‐Fc was used to test the potential binding of sCD155 present in supernatants to its receptor DNAM‐1 on NKL cells. Flow cytometry analysis demonstrated that both, ULBP2‐Fc and CD155‐Fc bound to NKL cells (Figure S3C). Although the addition of medium led to a slight reduction of ULBP2‐Fc binding, no further effect was seen by the culture supernatants, independently of their low or high amounts of sULBP2 or sCD155, respectively, strongly suggesting that the soluble ligands did not bind to their receptors on NKL cells.

In summary, although soluble ULBP2 was secreted in high amounts from cells infected with the ULBP2‐expressing virus variants, no negative impact was seen regarding NKG2D receptor modulation or recognition and killing of K562 target cells.

### 2.4 NK Cells Control Viral Spread with a Minor Impact of ULBP2

**FIGURE 3:**
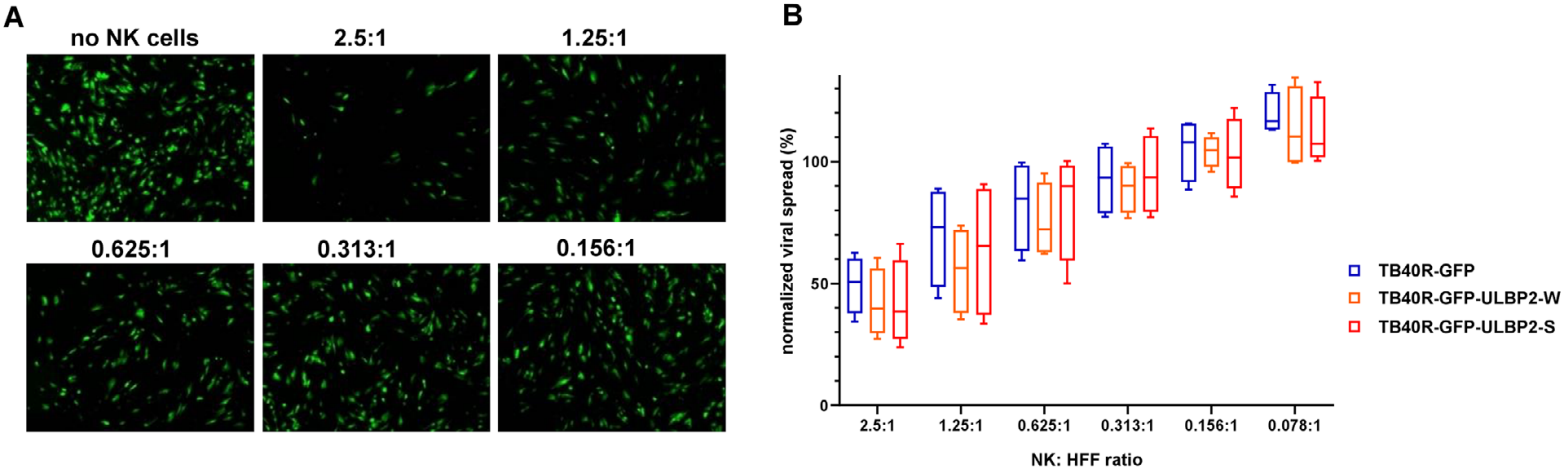
NK cell‐mediated control of viral spread. (A, B) HFF cultures were infected with GFP‐expressing variants of the different TB40R viruses at an MOI of 0.1 and co‐cultured with NK cells for 7 days at the indicated E:T ratios. Then, NK cells were cautiously taken off and GFP signals of infected cell cultures were measured. (A) Fluorescence‐microscopic images of HFF cultures 7 days after infection with the GFP‐expressing TB40R variant and incubation with NK cells at the indicated E:T ratios. (B) Viral spread (in %) was calculated by relating the GFP signals to those of HFF cultures kept without NK cells. The box‐whisker plots depict medians and the interquartile range of data for NK cells of four different donors (*n* = 4).

To assess the potential role of NKG2D activation in the control of viral spread, we adopted a virus dissemination assay (VDA) previously described by Chen et al. [33]. Therefore, new TB40R virus variants expressing GFP were constructed, which allowed for monitoring of viral spread by the quantification of GFP signals of infected cells. HFF were infected with the GFP‐expressing virus variants, that is, TB40R‐GFP, TB40R‐GFP‐ULBP2‐W, and TB40R‐GFP‐ULBP2‐S were comparable to non‐GFP‐expressing HCMV variants regarding their surface molecule expression (Figure S4A). For the VDA, these GFP‐expressing variants were used for infection of HFF at a low multiplicity of infection (MOI = 0.1) and co‐cultured at different effector‐to‐target (E:T) ratios with primary NK cells of 4 different donors for 7 days (Figure 3). The viral spread was calculated using the GFP signals of the cultures (w/o NK cells as 100% and % reduction in the presence of NK cells). The spread of the parental TB40R‐GFP virus was controlled by NK cells in an E:T ratio‐dependent manner, similar to those described by others [33, 34]. There was a trend toward slightly lower dissemination of both ULBP2‐expressing virus variants; however, it did not reach statistical significance, partly due to variations between the NK cell activity of different donors. When low doses of hIL‐15 (5 ng/mL) were added to the cultures to sustain NK cell activity, stronger inhibition of viral spread was observed, but again with little additional impact of ULBP2 expression, which was primarily seen at low E:T ratios (Figure S4B). Measuring the number of HFF target cells confirmed that the cytotoxic activity of NK cells was generally higher in the presence of hIL‐15 (Figure S4C,D). Altogether, our data indicate that the spread of TB40R virus variants was well‐controlled by NK cells, with a minor additional effect of ULBP2 expression.

### 2.5 Effect of Viral ULBP2 Expression on Receptor Modulation and Activation on NK Cells Subsets

**FIGURE 4:**
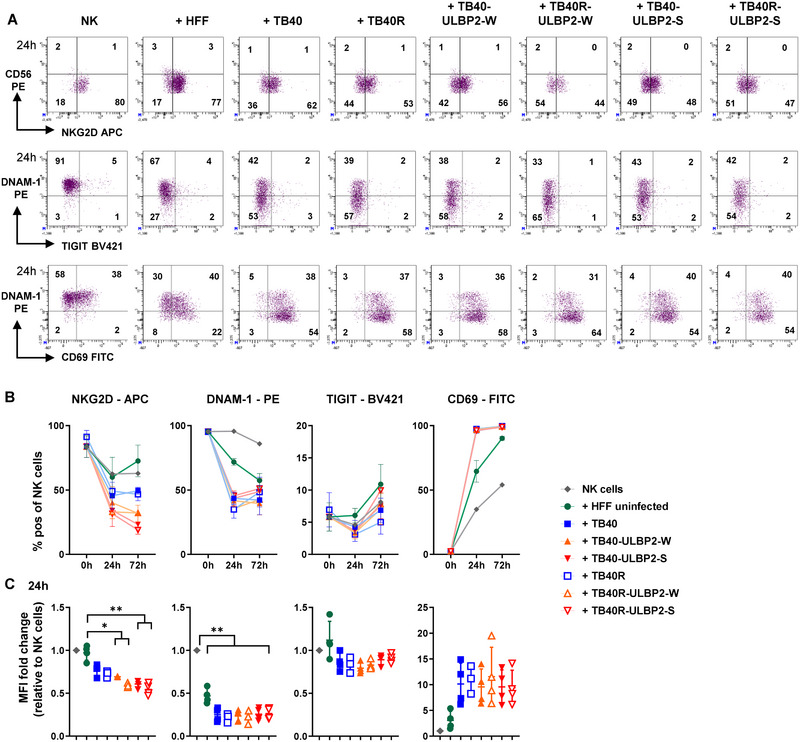
NK cell receptor modulation and activation upon co‐cultivation with HCMV virus‐infected HFF. PBMC obtained from healthy HCMV‐negative donors were co‐cultured with uninfected HFF or 4 dpi HCMV‐infected HFF. At 0, 24, and 72 h NK cells (gated by CD56^+^, CD3^−^; representative gating strategy is shown in Figure S5A) were evaluated by flow cytometry regarding the frequency and MFI of the indicated surface molecules. (A) Frequencies of NK cells (in %) of one representative donor after 24 h without (left) or with contact to uninfected and infected HFF (right). (B) Frequencies of NK cells (in %) at the indicated time‐points (mean ± SEM, *n* = 3–4). (C) MFI was normalized to MFI of NK cells that were not in contact with target cells and are depicted as fold change (mean ± SD, *n* = 3–4). Statistical analysis for (C) was performed by one‐way ANOVA mixed‐effects analysis with Tukey's multiple comparisons. *p < 0.05, **p < 0.01.

Next, we assessed with different assays whether the effects of the ULBP2‐NKG2D interaction could be detected at earlier time points and whether it is affecting also activation because the VDA captured only the overall outcome after 7 days. HFF infected with the different virus variants were co‐cultured with PBMC, and NK cells (defined as CD45^+^ CD56^+^ CD3^−^ cells; Figure S5C) were analyzed by flow cytometry after 24 and 72 h with respect to frequency and expression levels (MFI) of NKG2D. In addition to NKG2D, the activating and inhibitory receptors of CD155, DNAM‐1, and TIGIT, were also examined. Upon contact with infected cells, NKG2D was downmodulated and, hence, the proportion of NKG2D^+^CD56^dim^ NK cells was reduced (Figure 4A, upper panel). This effect was significantly influenced by the ULBP2 density, especially upon infection with the ULBP2‐S variants (Figure 4B,C). Thus, interactions of NKG2D on NK cells with the ULBP2 transgene expressed on infected HFF triggered NKG2D downregulation in a dose‐dependent manner.

Since both TB40 and TB40R variants influenced several other surface molecules, for example, CD155 (cf. Figure 1B,C), we included also DNAM‐1 and the inhibitory receptor TIGIT into the FACS panel. In response to virus‐infected cells, DNAM‐1 expression on NK cells was reduced as well as the proportion of DNAM‐1‐positive NK cells, arguing for a direct CD155‐DNAM‐1 interaction (Figure 4A–C). In contrast to the large DNAM‐1^+^ NK cell population (≥90%), only a small NK cell fraction was TIGIT^+^ with a moderate modulation upon contact with infected cells.

The capacity of infected cells to activate NK cells was quantified by induction of CD69, HLA‐DR, and CD25 as well as CD16 downregulation. While only a small fraction of NK cells showed upregulation of CD25 and HLA‐DR (Figure S6A–C), CD69 expression was strongly induced after 24 h and almost all NK cells were CD69^+^, following contact with virus‐infected cells (Figure 4B,C). The combination of DNAM‐1 with CD69 staining demonstrated that activation, that is, CD69 induction, was accompanied by DNAM‐1 downregulation (Figure 4A). In addition, in the course of NK cell activation, CD16 was downregulated on CD56^dim^ NK cells upon contact with infected cells (Figure S6A–C) despite the absence of human antibodies in the co‐cultures. Moreover, it is well known that HCMV infection can induce the expansion of NKG2C^+^ adaptive NK cells [35]. In our co‐culture experiments over 72 h, a moderate increase in NK cell populations expressing CD94 and NKG2A was observed; however, expansion of NKG2C^+^ NK cells was not detected (Figure S6A–C).

In conclusion, next to NKG2D receptor modulation dependent on ULBP2 density, contact with ULBP2‐HCMV variants resulted in further ligand–receptor interactions and activation of NK cells.

### 2.6 Enhanced NK Cell Cytotoxicity in Response to ULBP2‐HCMV Variants

**FIGURE 5:**
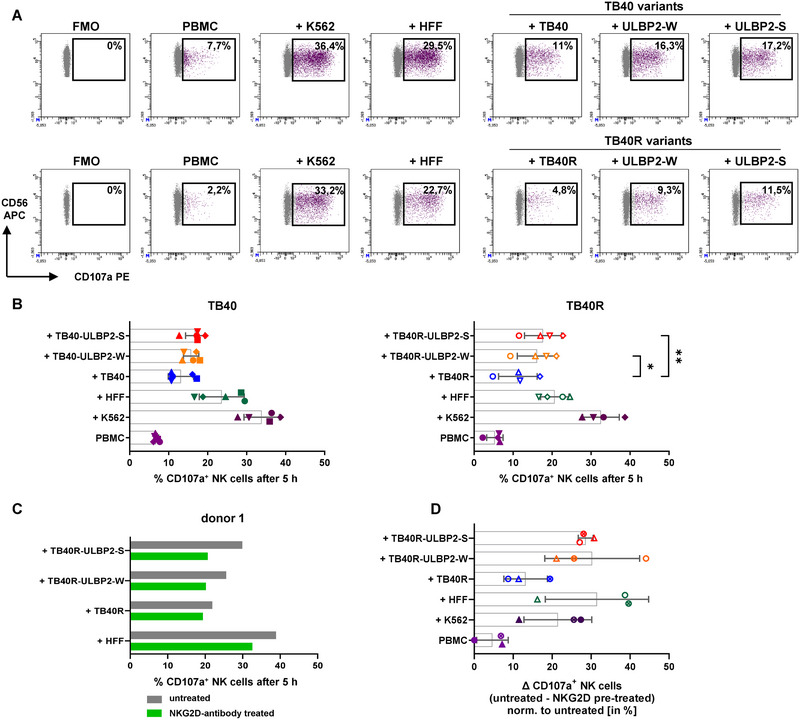
Degranulation capacity of NK cells in response to HCMV‐infected cells. 48 h IL‐2 prestimulated PBMC obtained from healthy HCMV‐negative donors were incubated with uninfected and 5 dpi HCMV‐infected HFF for 5 h to analyze the degranulation capacity of NK cells (gated by CD56^+^, CD3^−^; representative gating strategy is shown in Figure S5B). CD107a expression was measured by flow cytometry analysis. (A) Frequencies of CD107a^+^ NK cells of one representative donor, (B) frequencies of CD107a^+^ NK cells depicted for 4 or 5 different donors (mean ± SD, *n* = 4–5). Each specific symbol represented one donor. FMO control for CD107a was used to set the CD107a positive gate, and incubation with K562 cells served as positive control. (C, D) PBMC from three donors were pretreated with an unconjugated NKG2D antibody prior to degranulation assays and frequencies of CD107a^+^ NK cells were analyzed. As a control, PBMC were left untreated. Data of one representative donor are depicted (C); the two additional donors are shown in Figure S7D. Delta of CD107a^+^ NK cells between untreated and NKG2D mAb pretreated condition (untreated–NKG2D mAb treated) normalized to untreated shown in percentage (mean ± SD, *n* = 3) (D). Significance values for (B) were determined by using one‐way ANOVA with Tukey's multiple comparison test. *p < 0.05, **p < 0.01.

Finally, we assessed the impact of ULBP2 expression on NK cell‐mediated cytotoxicity measured by degranulation via CD107a surface staining of NK cells after 5 h co‐incubation with uninfected or infected HFF (gating strategy depicted in Figure S5B). Degranulation upon contact with HLA class I‐deficient K562 was used as a positive control (Figure 5A,B). Degranulation was markedly reduced upon contact with HFF infected with the parental TB40 and TB40R viruses compared with uninfected HFF, presumably due to low expression of activating ligands like ULBP2, ULBP1, or MICA (see Figure 1B,C; Figure S1C,D). Compared with TB40R‐infected cells, degranulation was, however, significantly increased upon contact of NK cells to infected cells with high ULBP2 expression, that is, following infection with TB40R‐ULBP2‐W or TB40‐ULBP2‐S. To further verify a ULPB2‐dependent enhancement of NK‐cell cytotoxicity, we preincubated PBMC with an unlabeled NKG2D mAb and thereafter added the target cells. Reduced binding of a labeled NKG2D mAb served as a control to confirm the effect of unconjugated NKG2D mAb preincubation (Figure S7A,B). Upon preincubation with NKG2D antibody, the beneficial NKG2D‐ULBP2 effect was no longer detectable (Figure 5C, Figure S7D), whereas preincubation with the isotype control Ab did not affect the percentage of CD107a^+^ degranulating NK cells (Figure S7C). Preincubation with a NKG2D mAb showed the strongest effect of reduction of CD107a^+^ NK cell frequencies when in contact with ULBP2‐expressing cells (Figure 5D). This confirms that the previously observed difference between WT and ULBP2 variants was due to the specific interaction between NKG2D and ULBP2.

Moreover, to exclude an impact of KIR/HLA class I interactions, for example, missing self‐recognition, we analyzed the proportions of KIR2DL/S1, KIR2DL/S2/3, and KIR3DL/S1 positive NK cells in different donors and their contribution to CD107a^+^ degranulation upon contact to infected cells with and without ULBP2 (Figure S8). Only small fractions of all degranulating CD107a^+^ NK cells were KIR‐positive indicating that the major NK cell fraction responding to ULBP2 was KIR‐negative. Thus, the specific interaction between NKG2D and ULBP2, rather than the influence of missing self‐recognition, was responsible for the differences in the presence of ULBP2.

Since CD8^+^ T cells also express NKG2D, we investigated whether T cells may contribute to NK cell degranulation. As expected, we did neither observe ULBP2‐dependent degranulation of CD8^+^ T cells nor degranulation by CD8^+^ T or CD4^+^ T cells in response to infected and uninfected HFF (Figures S5B and S9). Hence, the presence of T cells has no influence on NK cell degranulation in our experimental setting.

Taken together, the results were neither influenced by a KIR‐ligand mismatch, nor by the presence of T cells, but are the result of the specific interaction between NKG2D and ULBP2.

## 3 Discussion

Using novel HCMV variants, we could demonstrate that expression of the NKG2D‐L ULBP2 by promoters of different strengths resulted in a gradient of ULBP2 at the surface of infected HFF as well as differential shedding of soluble ULBP2 into cell culture supernatants. However, we did not observe the blocking effects of even high concentrations of sULBP2 on NK cell functions. To investigate whether ULBP2 expression on infected HFF can improve NK cell‐mediated viral control in vitro, 7‐day viral dissemination assays were performed. Viral spread in HFF cultures was inhibited by NK cells for all HCMV variants in an effector‐to‐target cell‐dependent manner. ULBP2 expression in infected HFF resulted in slightly improved control of dissemination for these variants. A significantly stronger impact of ULBP2 expression was detectable for NK cell degranulation, independent of KIR‐ligand mismatch, within 5 h upon contact with HFF infected with ULBP2‐W or ULBP2‐S variants. In detail, we could show that (1) HFF infected with wildtype TB40 or TB40R induced less NK cell activation and cytotoxicity due to lower NKG2D‐L expression compared with uninfected cells. (2) This resistance could be reverted in HFF infected with both ULBP2 variants due to high ULBP2 expression resulting in significantly stronger NK cell activation via NKG2D associated with enhanced cytotoxicity. Since the beneficial effect of enhanced ULBP2 expression was no longer detectable upon preincubation with NKG2D antibody, the increased cytotoxicity was demonstrated to be dependent on ULBP2. This ULBP2‐mediated cytotoxicity was consistent with a more intense NKG2D downregulation on NK cells upon interaction with ULBP2‐expressing HFF, depending on its expression level. Thus, the ULBP2‐mediated effect on NK cell activity is phase‐dependent with the strongest impact early after interaction with infected cells. Later, additional ligand‐receptor interactions, for example, CD155‐DNAM‐1, are likely to contribute to NK cell activity over time. Accordingly, multiple pathways contribute to viral control by NK cells, which are likely to overwrite the effect triggered initially by the ULBP2‐NKG2D axis.

This study extends previous analyses [20, 22, 25], which evaluated the feasibility of NKG2D‐L to attenuate CMV vaccine strains. In mouse models, viral expression of the RAE‐1γ ligand resulted in strong NK cell‐mediated control of this MCMV mutant, which was mainly driven by NKG2D signaling, although the contribution of another receptor could not be excluded [20, 22]. Our first analysis of a ULBP2‐expressing HCMV mutant in vitro pointed towards a similar NKG2D‐dependent inhibition of viral spread by human NK cells [25]. In the present study, we included also HCMV variants expressing ULBP2 either under the weak intrinsic UL16 or the strong MIEP promoter, also in the presence of all immune evasins in the TB40R variant. As expected, ULBP2 surface expression and shedding were significantly higher upon usage of the MIEP vs. UL16 promoter. Soluble NKG2D‐L like ULBP2 were described as immune escape mechanisms of viruses and tumors and the potential mechanisms are still discussed controversially between blocking or downregulation of NKG2D by soluble NKG2D‐L versus reduced surface levels of NKG2D‐L [31, 36, 37, 38, 39]. In several NK cell assays, we did not observe NKG2D downregulation on NK cells by sULPB2, interference with NKG2D interaction, or impaired cytotoxicity, which is in line with the data of Waldhauer and Steinle [31]. The simultaneously high surface and soluble ULBP2 levels argue for constant replenishment by newly synthesized molecules. Despite these in vitro findings, the role of sULBP2 in vivo cannot be completely excluded. Therefore, we favor the ULBP2‐weak variant as a vaccine candidate due to its similar capacity to activate NK cells but lower sULBP2 secretion compared with the ULBP2‐S variant. Nevertheless, in future work, we also plan to evaluate HCMV mutants that express other NKG2D ligands with lower shedding propensity [32, 40].

Our data confirm that multiple activating and inhibitory signals are integrated synergistically to trigger NK cell activation. First, both the TB40 and the TB40R viruses were highly efficient in HLA class I downregulation, abrogating the inhibitory signals for NK cells, known as missing self‐recognition. Simultaneously, all HCMV strains counteract NK cell activation by a targeted downregulation of ligands for activating receptors, for instance via UL16 [11, 29]. Therefore, the replacement of UL16 with ULBP2 was chosen as the integration site to allow high surface expression. The viral immune evasins not only reduce HLA class I surface expression but also affect the repertoire of presented viral peptides [41, 42, 43], which may lead to poor specific cytotoxic T‐cell responses. Hence, partial or complete deletion of US2‐11 immune evasins needs further investigation. As expected, stronger effects on HLA class I downregulation were seen for TB40R variants encoding all immune evasins compared with TB40 variants that retained only US10 and US11 but lacked the US2‐6 region. Both ULBP2‐S virus variants displayed even further reduced HLA class I levels, confirming our previous observation [25]. Besides ULBP2 expression by these variants, upregulation of other NKG2D‐L such as ULBP1 was observed due to UL16 deletion [19]. In contrast, MICA, a known target of US18, US20, UL142A, and UL148, was downregulated in all infected fibroblasts [16, 17, 18].

In addition, we investigated HLA‐E due to its role in the expansion of NKG2C^+^ adaptive NK cells upon HCMV infection [35, 44]. In our setting, HLA‐E expression was low and infection led to a further reduction due to the activity of immune evasins [30] and the absence of an HLA‐E stabilizing peptide [27] in the UL40 protein of our TB40 strains. Here, expansion of NKG2C^+^ NK cells was not observed, most likely due to HCMV‐negative donors and the short co‐culture period.

In parallel, the DNAM‐1/TIGIT ligand CD155 was examined, which was upregulated on all virus‐infected cells, since the TB40 and TB40R strains are deficient for the UL141 gene [14, 26]. Consequently, the activating DNAM‐1 receptor was downregulated on NK cells upon contact with infected cells. In contrast, the inhibitory receptor TIGIT was expressed only on small NK cell subsets without significant changes. Thus, CD155 modulation by HCMV seems to intensify DNAM‐1‐mediated signals rather than leading to TIGIT‐mediated inhibition. The simultaneous upregulation of the CD69 activation marker with DNAM‐1 downregulation supports this interpretation. The CD155‐DNAM‐1 axis was previously shown by Tomasec et al. [14] to be important for the regulation of NK cell function. Similarly, infection of monocyte‐derived dendritic cells with the TB40/E strain led to NK cell activation via DNAM‐1 [45]. The CD155‐DNAM‐1 axis may, therefore, be useful as an additional activating pathway for NK cell‐mediated control and HCMV attenuation.

Our results demonstrate simultaneous modulation of several ligands like ULBP2 and CD155, leading to activation via their receptors NKG2D and DNAM‐1, respectively, and HLA class I downregulation via immune evasins. With our ULBP2‐expressing mutants, the balance was shifted toward enhanced NK cell activation, which may harbor the risk of high attenuation with poor immunogenicity. Therefore, it will be necessary to investigate HCMV‐specific T‐cell responses in the future.

Taken together, we showed in this study that virally induced expression of NKG2D ligands and other activating NK cell ligands can be employed for strong NK cell‐mediated control of viral dissemination and attenuation of HCMV vaccine candidates. This provides also the basis for further refinement of HCMV vaccine developments, including the utilization of other NKG2D‐L (ULBP1, MICA, or MICB) and DNAM‐1 ligands and the modulation of HLA class I expression.

## 4 Data Limitations and Perspectives

In this proof‐of‐concept study, virus‐infected fibroblasts and NK cell donors could only be partially matched for HLA and KIR ligands. In the future, validation of ULBP2‐expressing HCMV variants in a matched autologous setting is planned using dendritic cells infected with the HCMV variants. Moreover, the potential of other ligands for activating NK cell receptors such as CD155 or other NKG2D ligands will be studied, especially since the NKG2D‐L differ in some of their properties that affect signaling via NKG2D.

## 5 Material and Methods

### 5.1 Cell Lines and Cell Culture

Human foreskin fibroblasts (HFF; Merck Millipore) were maintained in DMEM (Thermo Fisher) supplemented with 10% FCS (Gibco/Bio&Sell), 100 U/mL penicillin (Gibco), 100 µg/mL streptomycin (Gibco), and 20% FibroGro containing supplements (Merck Millipore). RPMI1640 medium (Thermo Fisher) supplemented with 100 U/mL penicillin, 100 µg/mL streptomycin, 2 mM L‐glutamine (Gibco), and 1 mM sodium pyruvate (Gibco) was used for cultivation of NKL cells [30], NK92 (ACC488) and K562 (ATCC). This media was additionally supplemented with 15% FCS and 200 U/mL rhIL‐2 (Peprotech) for NKL cells, with 15% FCS, 10% human serum (PAN‐Biotech) and 200 U/mL rhIL‐2 for NK92 (provided by Eric Vivier, INSERM, Marseille), and 10% FCS for K562.

### 5.2 PBMC Isolation and NK Cell Enrichment

Peripheral blood mononuclear cells (PBMC) were isolated from EDTA blood of HCMV‐negative healthy donors (MHH ethical vote n° 968–2011) by Biocoll density‐gradient centrifugation (Bio&Sell) and either used directly for isolation of NK cells or after cryopreservation. Primary NK cells were isolated from PBMC by negative selection, using the NK isolation kit (Miltenyi Biotec) with either an autoMACS Pro Separator or manual separation using MS columns (both Miltenyi Biotec) according to the manufacturer's instructions.

### 5.3 Generation of ULBP2‐Expressing HCMV Variants

HCMV variants expressing the NKG2D‐L ULBP2 were based on the BAC‐cloned HCMV strains TB40 [26] and TB40R [27]. By replacing the ORF UL16, ULBP2 was either expressed under the UL16 promoter (ULBP2‐W) or the strong MIEP promoter (ULBP2‐S). In addition, for viral dissemination assays, TB40R‐ULBP2‐W/‐S variants were further equipped with a GFP reporter gene. A detailed description can be found in the supporting information.

### 5.4 Infection of HFF Cultures

HFF at ∼80% confluence were inoculated with viruses at indicated MOI for 3 h, followed by medium replacement. Alternatively, infection was performed by mixing uninfected HFF with cells infected for 7 days at a ratio of 4:1 and the addition of 10% supernatant of the 7‐dpi culture.

### 5.5 Flow Cytometry

Flow cytometry was performed according to recommended guidelines [46]. To discriminate between live and dead cells, the yellow fluorescent reactive dye (Thermo Fisher) was used following the manufacturer's instructions. Cells were stained with the respective antibodies (Tables S1 and S2) in FACS buffer (0.1% NaN_3_, 1% FCS in PBS) at 4°C for 30 min. If secondary antibodies (Table S3) were needed, incubation was done for 30 min at RT. Prior to data acquisition using an LSRII cytometer (BD Biosciences), cells were fixed with 1% PFA in PBS. Data were analyzed using FACSDiva software v8.0.1 (BD Biosciences) or FlowJo v10.8.1 (Treestar).

### 5.6 Quantification of Soluble ULBP2

Amounts of soluble ULBP2 (sULBP2) in cell culture supernatants were quantified with Human ULBP‐2 DuoSet ELISA (R&D Systems; cat. DY1298) according to the manufacturer's instructions. Supernatants taken from uninfected and TB40(R)‐infected cell cultures were diluted 1:2 with the provided reagent diluent, and those taken from TB40(R)‐ULBP2‐W and from TB40(R)‐ULBP2‐S infected cells were diluted 1:100 and 1:3200, respectively. sULBP2 concentrations (ng/mL) were calculated using standard curves.

### 5.7 Analysis of the Effect of Soluble ULBP2 on Killing Ability and Receptor Modulation of NK Cell Lines

NKL and NK92 cells were seeded at a density of 200,000 cells/mL, and one day later incubated for 2 h with supernatants of 6–7 dpi infected or uninfected HFF cultures at RT. As a control, the unconjugated NKG2D antibody (5 µg/mL; R&D Systems) was used to analyze the effect of NKG2D receptor blocking. K562 cells were labeled with CFSE (BD Bioscience, cat. 51–9010817, labeling was for 7 min with a conc. of 1 µM), and added to the preincubated NKL and NK92 cells at an E:T of 5:1, followed by overnight incubation. Then, cells were stained with the viability dye, an NKG2A‐PE‐Cy7 Ab (Beckman Coulter, cat. B10246) for gating strategy and with the NKG2D‐APC Ab (Thermo Fisher; cat. 17‐5878‐42) to provide information about the NKG2D receptor modulation or blocking. Anti‐Mouse Ig ĸ Beads (BD Bioscience) were stained with CD45‐AF700 (Biolegend,) at 4°C for 30 min. The addition of the labeled beads to the cells allowed us to calculate the relative number of living K562 cells, the number of total cells, and the killing rate. The following calculation formula was used:

$$rel.K562=\frac{(K562\;\mathrm{events}\;\times \;\mathrm{fixed}\;\mathrm{bead}\;\mathrm{number})}{\mathrm{sample}\;\mathrm{bead}\;\mathrm{number}}.$$

### 5.8 Quantification of Soluble NK Cell Ligands by Luminex‐Based Multiplex Assays

Soluble NK cell ligands (including NKG2D‐L) present in cell culture supernatants were quantified by using either an Immuno‐Oncology Checkpoint 14‐Plex Human ProcartaPlex Panel 2 (Thermo Fisher) or a Human ProcartaPlex Mix&Match 13‐plex (Thermo Fisher). All luminex‐based multiplex assays were performed according to the manufacturer´s instructions. Samples were acquired by the Bio‐Plex machine and the Bio‐Plex‐Manager v6.0 software, and data was analyzed using the Bio‐Plex Manager v6.1 software.

### 5.9 Co‐Culture of Infected HFF and PBMC

HFF infected for 4 days or uninfected HFF were co‐cultured with allogeneic PBMC at a 12:1‐16:1 effector‐to‐target ratio in supplemented PRMI and 10% FCS. As control, PBMC were kept in the absence of target cells. At time points 0, 24, and 72 h upon co‐culture, PBMC were surface stained with antibodies (Table S1) and analyzed by flow cytometry. An exemplary gating strategy is depicted in Figure S5A. After 24 hours of co‐culture, 200 U/ml rhIL‐2 was added to the medium for the further course of the culture.

### 5.10 CD107a Degranulation Assay

PBMC were seeded at a density of 1.5 × 10^6^ cells/mL in supplemented RPMI containing 100 U/mL rhIL‐2 and incubated for 48 h. Then, PBMC were added at an effector‐to‐target ratio of 1:1.5 either to uninfected HFF or to cells infected for 5 days. Spontaneous degranulation was assessed with PBMC seeded without target cells. The CD107a‐PE Ab (BD Bioscience) or (for degranulation assays in combination with KIR expression analysis) the CD107a‐APC Ab (BD Bioscience), was added immediately and monensin (Sigma) after 1 h. After an additional 4 h, cells were analyzed by flow cytometry with live/dead discrimination and staining for lineage markers, and KIR antibodies if necessary (antibodies in Table S1). An exemplary gating strategy is depicted in Figure S5B, S8A (for KIR analysis). For experiments using NKG2D antibody preincubation, PBMCs were incubated with 10 µg/mL NKG2D antibody (unconjugated; BD Pharmingen) or isotype antibody control (MOPC21, mαhIgG1, kappa, Sigma) for 45 min at room temperature followed by a washing step, to remove unbound NKG2D antibody, prior to the addition of target cells. An exemplary gating strategy for these NKG2D Ab preincubation experiments is depicted in Figure S5B.

### 5.11 In Vitro Viral Dissemination Assay

HFF were seeded into 96‐well plates at a density of 1 × 10^4^ cells per well and infected one day later at an MOI of 0.15. After another 3 h, NK cells were added at indicated E:T ratios. If indicated, cells were cultivated in the presence of hIL‐15 (5 ng/mL; Peprotech). After 7 days of incubation, the GFP signals of the HFF cultures were measured using a Cytation 3 cell imaging multimode plate reader (Biotek). Subsequently, cells were fixed with methanol and stained with DAPI. Images of the DAPI‐stained cells were used to assess the cell numbers by the CellProfiler program (version 4.1.3, http://cellprofiler.org/; NIH, USA).

### 5.12 Statistical Analysis

Statistical analysis of the data was performed with the tests indicated in the figure legends by using GraphPad Prism v7.05 or v9.5.0. Differences were considered statistically significant for *p < 0.05.

## Author Contributions

Greta Meyer performed experiments, analyzed data, and wrote major parts of the manuscript. Anna Rebecca Siemes performed experiments, analyzed data, and supported writing of the manuscript. Jenny F. Kühne performed experiments and helped analyze the data and writing of the manuscript. Irina Bevzenko performed experiments, analyzed data, and supported writing the revised manuscript. Viktoria Baszczok performed experiments. Jana Keil, Kerstin Beushausen, and Karen Wagner supported experiments, and Lars Steinbrück sequenced viral genomes and performed sequence analysis. Christine S. Falk and Martin Messerle supervised the work, designed experiments, analyzed data, and wrote the manuscript. All authors agreed with the content of the manuscript and approved the final version.

## Ethics Approval

All participating subjects provided written informed consent and the ethics committee of Hannover Medical School approved the study (no. 968–2011).

## Conflicts of Interest

The authors declare no conflicts of interest.

### Peer Review

The peer review history for this article is available at https://publons.com/publon/10.1002/eji.202451266

## Supporting information



Supporting Information

## Data Availability

The data that support the findings of this study are available from the corresponding author upon reasonable request.
