# Supplementary material for: HCMV Variants Expressing ULBP2 Enhance the Function of Human NK Cells via its Receptor NKG2D
Source: Eur J Immunol. 2025 Feb 11;55(2):e202451266. doi: 10.1002/eji.202451266 (PMC11811812; doi:10.1002/eji.202451266)
Supplement: Supplementary file 1 — Supporting Information [file EJI-55-e202451266-s001.pdf]

HCMV variants expressing ULBP2 enhance the function of human NK cells via its receptor NKG2D

Greta Meyer[1§], Anna Rebecca Siemes[2§], Jenny F. Kühne[1], Irina Bevzenko[1], Viktoria Baszczok[1], Jana Keil[1], Kerstin Beushausen[1], Karen Wagner[2], Lars Steinbrück[2], Martin Messerle[2&], Christine S. Falk[1,3&]

[1] Institute of Transplant Immunology, Hannover Medical School, Hannover, Germany
[2] Institute of Virology, Hannover Medical School, Hannover, Germany
[3] German Center for Infection Research (DZIF), TTU-IICH (Infection of the immunocompromised host), Hannover/Braunschweig, Germany

[§] shared authorship [&] joint senior authors

Correspondence:

Christine S. Falk, PhD.
Institute of Transplant Immunology
Hannover Medical School, MHH,
Carl-Neuberg-Str. 1
30625 Hannover
Germany
Phone: +49 511 532 9745
Fax: +49 511 532 8090
e-Mail: falk.christine@mh-hannover.de

Supporting information – Methods

Generation of ULBP2-expressing HCMV variants

Growth curve analysis

Binding ability of soluble ligands to NKL cells

List of Supplementary Figures

Figure S1 Further characterization of ULBP2-expressing viruses.

Figure S2 Shedding of additional NK cell ligands.

Figure S3 Fusion protein binding in the presence of sULBP2 and sCD155

Figure S4 Control of viral spread by NK cells kept in the presence of hIL-15 and cytotoxic effect of NK cells.

Figure S5 Gating strategy of NK cells in coculture experiments and degranulation assays.

Figure S6 Modulation of additional NK cell receptors and surface markers upon co-culture with HCMV-infected HFF

Figure S7 Pre-incubation of NK cells with NKG2D antibody prior to degranulation assays

Figure S8 KIR expression pattern on degranulating CD107a^{+} NK cells

Figure S9 Degranulation capacity of CD8^{+} and CD4^{+} T cells in response to HCMV-infected cells

List of Supplementary Tables

Table S1 Primary conjugated antibodies

Table S2 Primary unconjugated antibodies

Table S3 Secondary antibodies

Supporting information – References

Supporting information – Methods:

Generation of ULBP2-expressing HCMV variants

The TB40-ULBP2 virus (TB40-ULBP2-S) was previously described [1]. The TB40-ULBP2-W variant was constructed by red-α, -β, -γ-mediated mutagenesis of the BAC cloned TB40-BAC4 genome [2] using a DNA fragment carrying the ULBP2 ORF and a kanamycin resistance gene (KanR), which was PCR amplified with primers UL16-neu.for (5´-CTGACGTAGGTACCGACTGGGGTCAAAAGCCTGGGTACTTCCAC-CATGGCAGCAGCCGCC-3´) and UL16.rev (5´-CTTATAGCAGCGTGAACGTTGCA-CGTGGCCTTTGCGGTTATC-CGTTCAGGAACACTTAACGGCTGA-3´) and the ULBP2 template plasmid [1]. KanR was subsequently excised by FLP recombinase. Mutagenesis of the TB40R BAC [3] was performed by *en passant* mutagenesis in *E.coli* GS1783 as described [4]. In brief, the ULBP2 ORF was amplified with primers MM005 (5'-GAGCTCCTCGCTGCAGCCGTAGAACGCAGAGCTC-3') and MM006 (5'-AGCTGCAATAAACAAGTTAACATAGGAACTTCAGATCCCTCTC-3') using an ULBP2 cDNA clone as template (Open Biosystems; Genbank accession number: BC034689) and cloned into PstI-HpaI cleaved plasmid pMCMV3 {Marquardt et al., 2011}, downstream of the mouse CMV major-immediate early promotor sequence (nucleotides 182849 to 183094 of the MCMV Smith Strain (Genbank accession number NC004065.1) by applying Gibson-Assembly cloning (NEB). A KanR cassette was amplification with primers MM007 (5'-TCAGTTTCGATGGGCAGA-TCTTCCTCCTCTTTGACTCAGAGACGCATCGTGGCCGGATC-3') and MM008 (5'-GAGTCAAAGAGGAGGAAGATCTGCCCATCGAAACTGAACTGCGTGACCACGTC-GTGGAATGC-3') and inserted into the BglII site of the obtained ULBP2 plasmid. For BAC recombineering, the MIEP-ULBP2-KanR and ULBP2-KanR fragments were PCR-amplified with primers UL16.fw (5'-GACACCGGGCTCCATGCTGACGTA-GGTACCGACTGGGGTCAAAAGCCTTTAAACGGTACTTTCCCATAGC-3') and UL16rev.new (5'-GCAGCGTGAACGTTGCACGTGGCCTTTGCGGTTATCCGTTC-AGCTTTATTTGTAACCATTATAAGCTGC-3') and primers UL16-neu.for and UL16rev.new, respectively. PCR products were used to electroporate recombination-proficient GS1783 *E.coli* bacteria containing the BAC TB40R [4]. The KanR cassette was excised by homologous recombination initiated by I-SceI mediated cleavage within the KanR gene. Correct insertion of the transgene was verified by restriction analysis of the BAC DNA and sequencing of the respective region. For generation of GFP-expressing TB40R virus variants, a PCR fragment encompassing the MIEP and

the mGFP ORF with inserted KanR cassette was generated using primers TB40-RL13-MIEP.for (5′-ACAACATCCGAAGAAACATCAATGCCCATTAACCGAAATCCAACAA-CGTTTAAACGGTACTTTCCCATAGCTG-3′) and TB40R-RL13-mGFP.rev (5′-GAAACATATTATTGGCTAAAAAGAAAAGCAAAAGTTTATTGGTGTGCATGTTACT TGTACAGCTCGTCCAT-3′) as recently described [5] and used to modify the respective BAC-cloned genomes.
Infectious virus was recovered by transfecting HFF cells with purified BAC DNA using the adenofection technique [6]. The integrity of the viral genomes was verified by Illumina sequencing. Production of virus stocks and virus titration by plaque assay were done as described previously [3].

Growth curve analysis

For growth curve analysis, HFF seeded in 6-well plates were infected with an MOI of 0.1. An aliquot of the inoculum was stored as day-0 sample. Supernatants of triplicates were collected at day 1 and then every second day until complete cell lysis was visible. Samples were stored at -80°C until viral titers (PFU/ml) were determined by plaque assay.

Binding ability of soluble ligands to NKL cells

Binding of sULBPP2 and soluble CD155 (sCD155) to the respective receptors was checked with a competition assay using the culture supernatants and chimeric recombinant human ULBP-2 Fc (R&D Systems; Cat. 1298-UL) or recombinant human CD155/PVR Fc fusion proteins (R&D Systems, Cat. 9174-CD). In brief, NKL cells were preincubated for 45 min at RT with supernatants of uninfected HFF or HFF cultures infected for 6-7 days. Afterwards, either the hULBP-2 Fc or the hCD155-Fc were added (at 5 µg/ml) for 30 min at RT, followed by staining with a secondary antibody and flow cytometric analysis.

Supplementary Figures

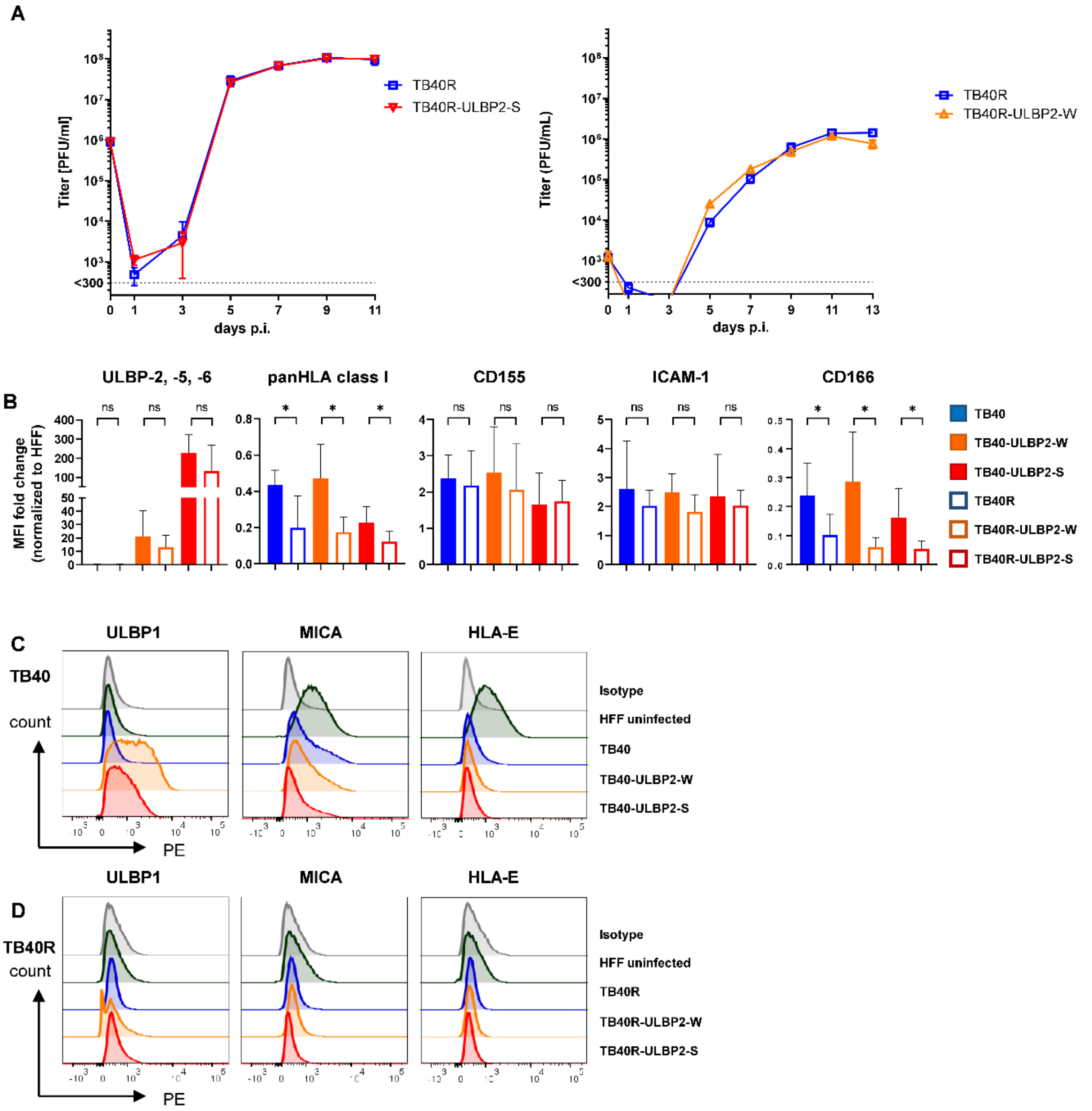

Supplementary Figure 1 Further characterization of ULBP2-expressing viruses. (A) Comparison of the growth kinetics of the ULBP2-expressing virus variants and of the parental virus. HFF were infected at an MOI of 0.1 (PFU/cell) and titers of infectious virus in the culture supernatants at the indicated days post infection (p.i.) were determined by plaque assay. **(B)** Comparison of the TB40 and TB40R mutants using fold changes shown in Figure 1B, C. Significance values were determined by using unpaired t-tests. p<0.05 (*), p<0.01 (**), p<0.001 (***), p<0.0001 (****). **(C, D)** Uninfected HFF and cells infected for 4 days with the indicated viruses were analyzed by flow cytometry regarding the expression of surface molecules. Median fluorescence intensities (MFI) are represented as overlays and as ΔMFI fold change (MFI – isotype) normalized to MFI of uninfected HFF (mean ± SD, n=3-4). Statistical analysis was performed by one-way ANOVA with Tukey´s multiple comparison test. p<0.05 (*), p<0.01 (**), p<0.001 (***), p<0.0001 (****).

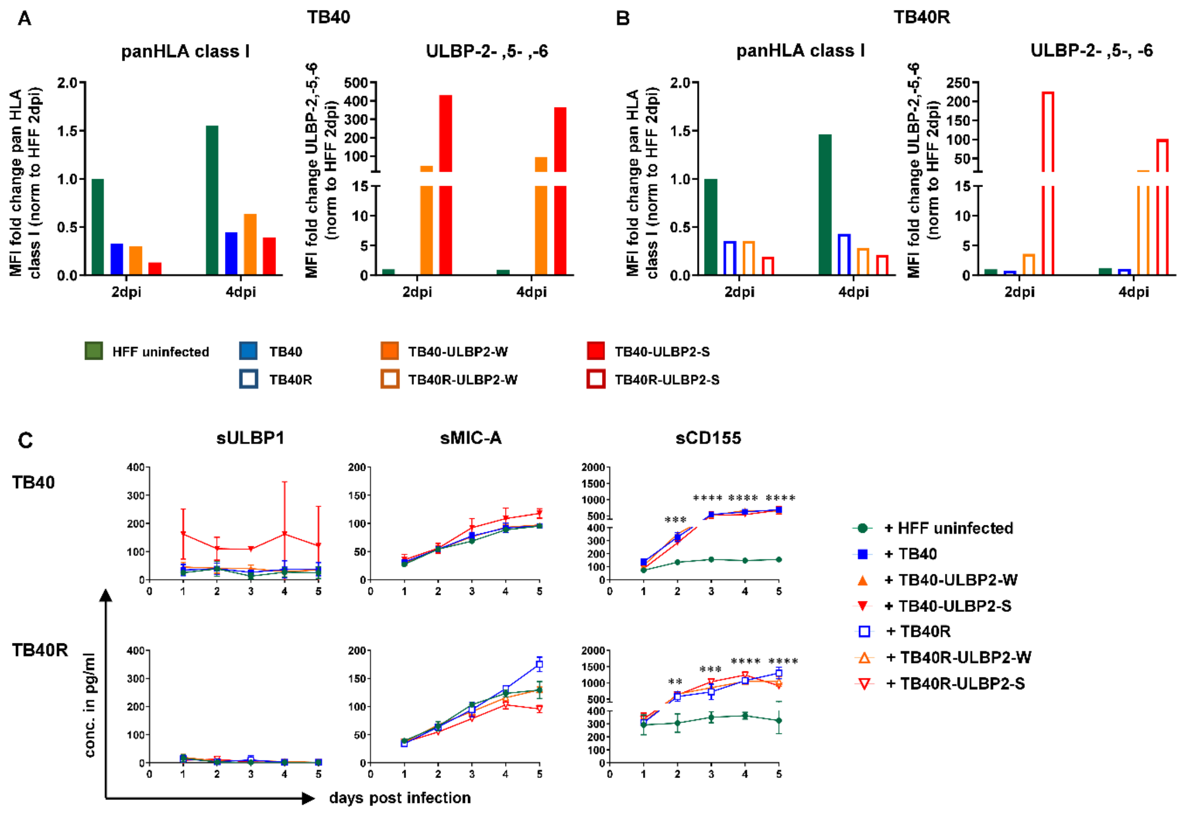

Supplementary Figure 2 Shedding of additional NK cell ligands. (A, B) In parallel to the analysis of NK cell ligand shedding over a 5-day period, uninfected and HCMV-infected HFF were analyzed by flow cytometry at day 2 and 4 p.i. regarding the surface expression of HLA class I and ULBP-2, -5, -6. ΔMFI fold change was calculated by normalization to MFI of uninfected HFF at day 2. **(C)** Luminex-based multiplex analysis was performed to quantify the concentration (pg/ml) of the indicated soluble NKG2D ligands and of CD155 (n=3 biological triplicates per condition, depicted as mean ± SD). Statistical analysis was performed by two-way ANOVA with Tukey´s multiple comparison test. $p<0.05$ (*), $p<0.01$ (**), $p<0.001$ (***), $p<0.0001$ (****).

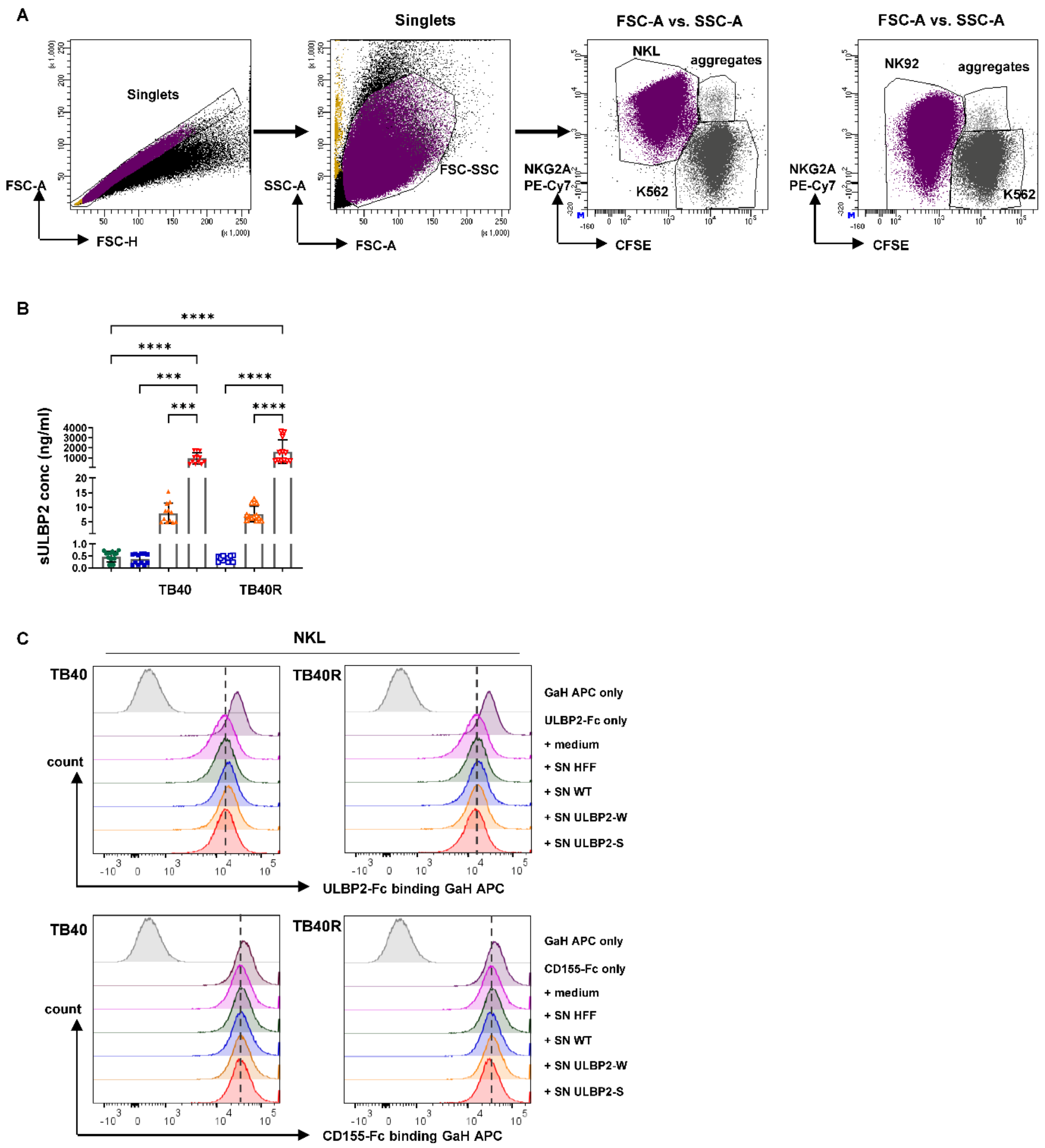

Supplementary Figure 3 Fusion protein binding in the presence of sULBP2 and sCD155. (A) Gating strategy used for the killing assays performed with NKL/NK92 and K562 cells (shown in Figure 2C-E). NKL and NK92 cells (both CFSE^{-}-negative) with high and intermediate expression of NKG2A are depicted in purple, K562 cells were discriminated by CFSE positivity and negativity for NKG2A (black). Synapse formation between NK cell lines and K562 target cells is referred as aggregates (grey). **(B)** Amount of soluble ULBP2 in supernatants of cultures used for killing assays were analyzed by ULBP2-ELISA (mean ± SD, n=4-5 in technical triplicates). Filled symbols represent data for TB40-variants, empty symbols for TB40R variants according to the color code **(C)** NKL cells were first supplied with supernatants of the indicated cell cultures, and subsequently incubated with recombinant human ULBP2-Fc and CD155-Fc fusion proteins and analyzed by flow cytometry to measure whether the presence of sULBP2 and sCD155 (in the supernatants) affects the fusion protein-binding.

Dashed line indicated the peak of fusion protein binding in the absence of culture supernatants. Statistical analysis **(B)** was done by one-way ANOVA with Tukey´s multiple comparison test. $p<0.05$ (*), $p<0.01$ (**), $p<0.001$ (***), $p<0.0001$ (****).

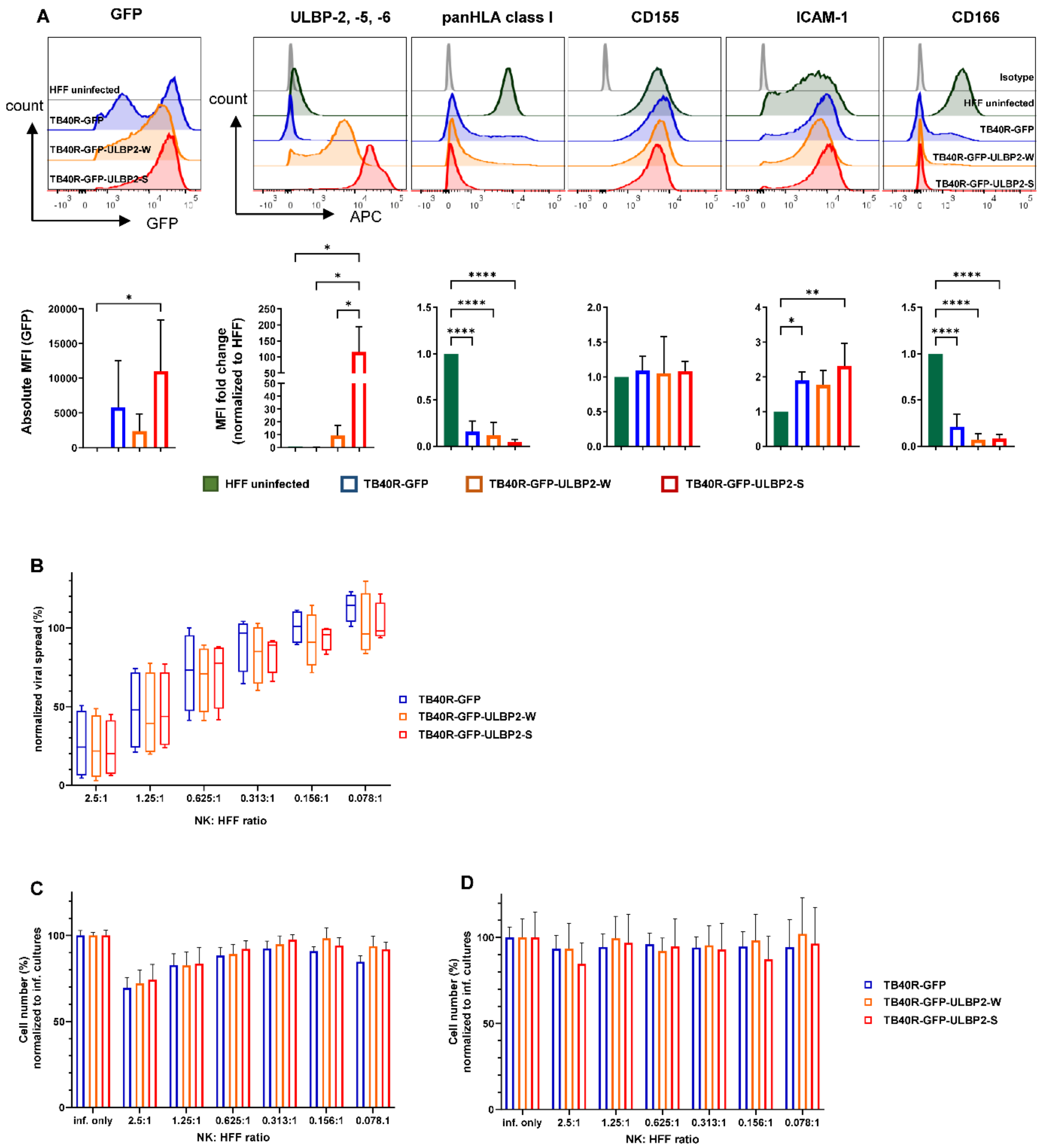

Supplementary Figure 4 Control of viral spread by NK cells kept in the presence of hIL-15 and cytotoxic effect of NK cells. (A) HFF infected with the different GFP-expressing HCMV variants for 4 days were analyzed by flow cytometry for expression of the indicated surface molecules. Absolute MFI of GFP signal is depicted (mean ± SD, n=2-5). MFI of surface molecule expression are represented as overlays and as ΔMFI fold change (MFI – isotype) normalized to MFI of uninfected HFF (mean ± SD, n=3-4). Statistical analysis was performed by one-way ANOVA with Tukey´s multiple comparison test, and statistical significance is indicated as * $p<0.05$, ** $p<0.01$, *** $p<0.001$, **** $p<0.0001$. **(B-D)** GFP expressing HCMV variants as described in Figure 3 were cocultured with NK cells in the presence of hIL15 (5 ng/ml) at the indicated E:T ratio. **(B)** Viral spread (in %) calculated by measuring GFP signals of the cultures at day 7 p.i. and relating them to GFP signals of cultures kept without NK cells (infection only). Boxes depict medians and interquartile ranges. **(C, D)** Cell numbers of infected

HFF cultures and co-cultured with NK cells either w/o **(C)** or with hIL15 **(D)** were counted on day 7 p.i. following DAPI staining and depicted in relation to cell numbers of cultures without NK cells (in %; mean + SEM).

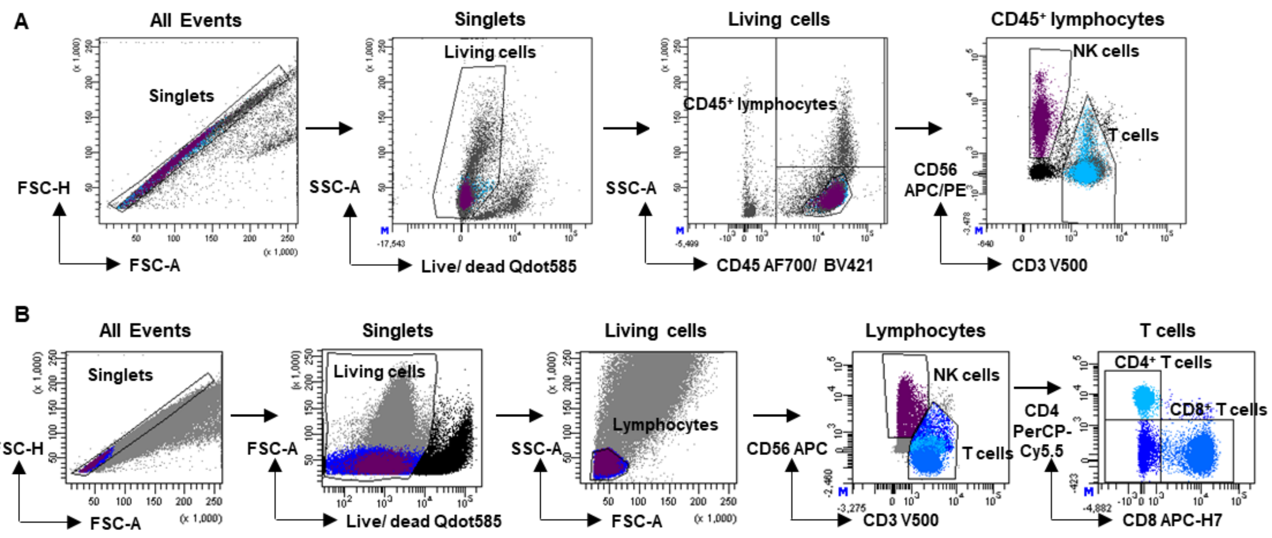

Supplementary Figure 5 Gating strategy of NK cells in coculture experiments and degranulation assays. (A) Representative gating strategy for NK cells in coculture experiments (singlets, alive, $CD45^+$ lymphocytes, $CD56^+$, $CD3^-$). **(B)** Representative gating strategy applied for degranulation assays. NK cells were defined as $CD56^+CD3^-$. T cells ($CD56^-$ $CD3^+$) were further subdivided according to CD4 or CD8 expression. For degranulation experiments with simultaneous analysis of KIR expression, please see **Supplementary Figure 8**.

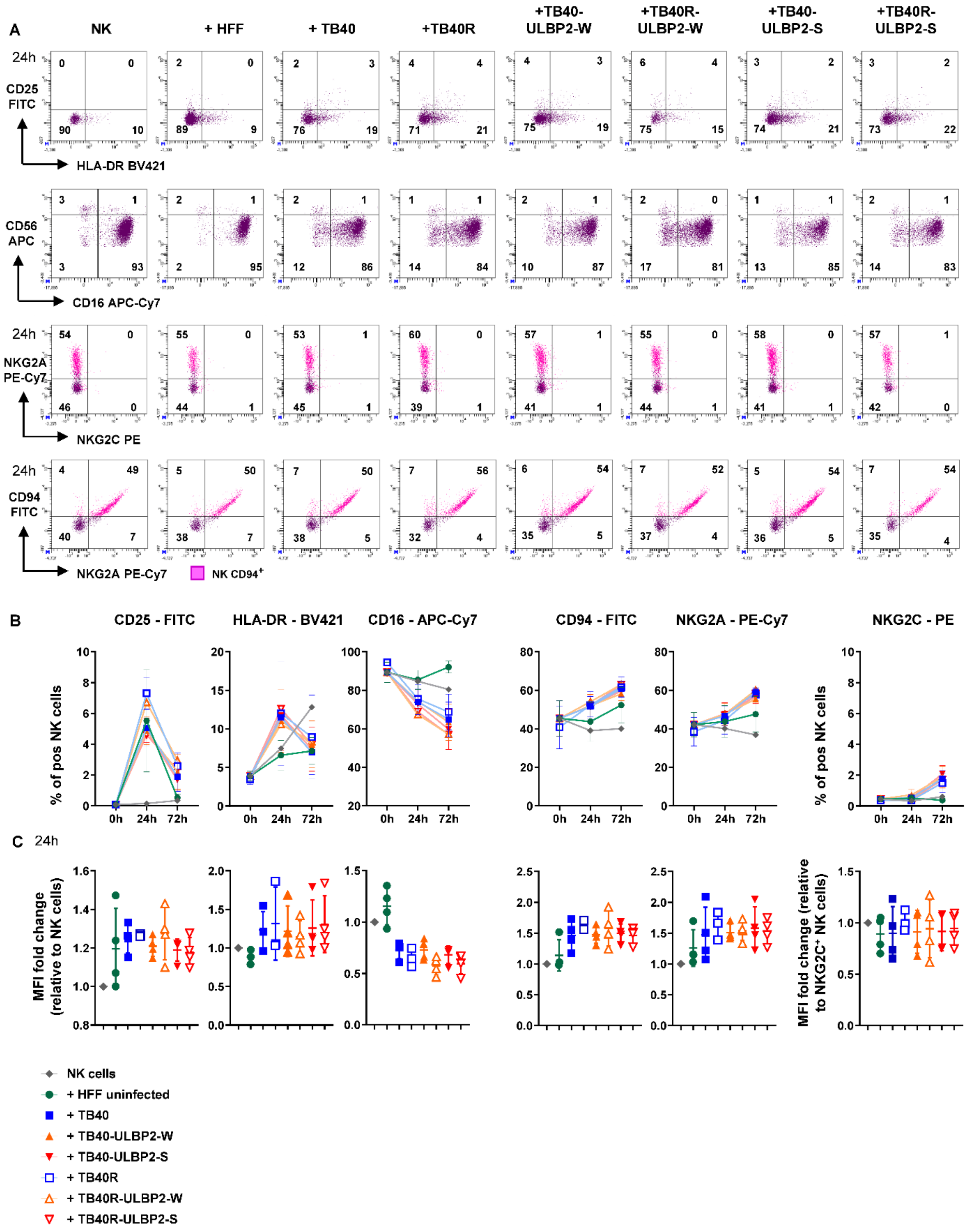

Supplementary Figure 6 Modulation of additional NK cell receptors and surface markers upon co-culture with HCMV-infected HFF. Allogeneic PBMC obtained from healthy HCMV-negative donors were co-cultured with uninfected HFF or HFF infected with the indicated viruses for 4 days. At 0, 24 and 72 h of co-culture NK cells were analyzed by flow cytometry (gated by CD56^{+}, CD3^{-}; representative gating strategy shown in Supplementary Figure 5A) regarding the frequency and MFI of expressed molecules. **(A)** Frequencies of NK cells (in %) of one representative donor expressing the indicated molecules without and with contact to uninfected or infected HFF after 24

h of co-culturing. **(B)** Frequencies of NK cells (in %) expressing the indicated markers over all time-points (mean ± SEM, n=3-4). **(C)** MFI normalized to MFI of NK cells that were not in contact with target cells depicted as fold change (mean ± SD, n=3-4). For NKG2C, MFI fold changes were normalized to $NKG2C^{+}$ NK cells not in contact with target cells (gray) (mean ± SD, n= 3-4).

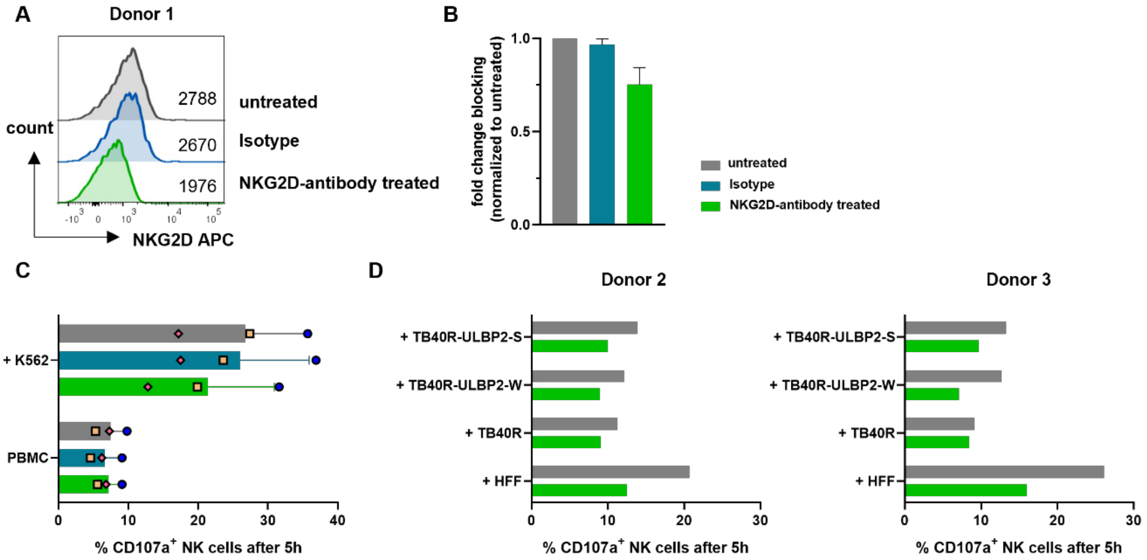

Supplementary Figure 7 Pre-incubation of NK cells with NKG2D antibody prior to degranulation assays. Following pre-stimulation for 48 h with IL-2, PBMC obtained from three healthy HCMV-negative donors were pre-incubated with 10ug/ml unconjugated NKG2D antibody or isotype antibody control (MOPC21, mIgG1, kappa) or left untreated as a control. After pre-incubation, uninfected and 5 dpi HCMV-infected HFF (TB40R variants) were added to the PBMC for 5 h to analyze the degranulation capacity of NK cells (gated by $CD56^{+}CD3^{-}$; representative gating strategy shown in Supplementary Figure 5B) by CD107a expression. **(A, B)** NKG2D antibody binding capacity by NK cells after pre-incubation with an unconjugated NKG2D antibody was analyzed by staining pre-incubated PBMC with a fluorochrome-conjugated NKG2D antibody, exemplarily shown with histogram overlays and MFI for one donor in **(A)** and normalized to untreated NK cells for all three donors in **(B)** (depicted as fold change, mean ± SD, n=3). **(C)** Frequencies of $CD107a^{+}$ NK cells that remained untreated (grey) or were pre-incubated with either the isotype antibody control (blue) or the NKG2D antibody (green) for the negative control (PBMC alone) and the positive control (+ K562). The three donors are represented by different symbols. **(D)** Frequencies of untreated or NKG2D-antibody treated $CD107a^{+}$ NK cells upon contact with uninfected or HCMV-infected HFF for the donors 2 and 3.

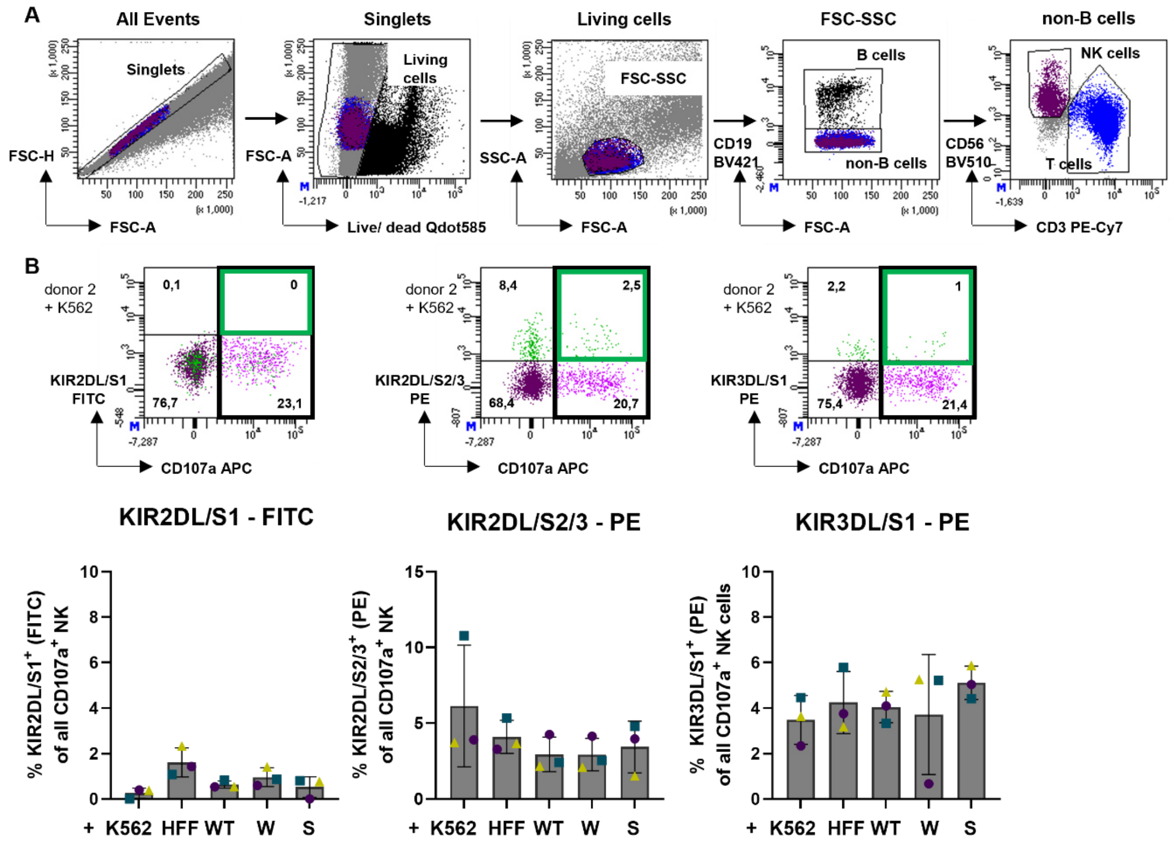

Supplementary Figure 8 KIR expression pattern on degranulating CD107a$^+$ NK cells. PBMC obtained from three healthy HCMV-negative donors were pre-stimulated for 48 h with IL-2 and subsequently added to either uninfected or 5 dpi HCMV-infected HFF (TB40R variants) for 5 h to analyze for KIR expression by degranulating NK cells. **(A)** Representative gating strategy with NK cells defined as CD19$^-$CD3$^-$CD56$^+$. **(B)** Frequencies of CD107a$^+$ NK cells expressing the respective KIR receptor is exemplarily shown in the upper row (donor 2 [blue square] with K562) and pooled for the three donors in the lower row (mean ± SD, n=3). Each symbol represents one donor. Note that the monoclonal KIR antibodies cannot distinguish between activating and inhibitory KIR receptors.

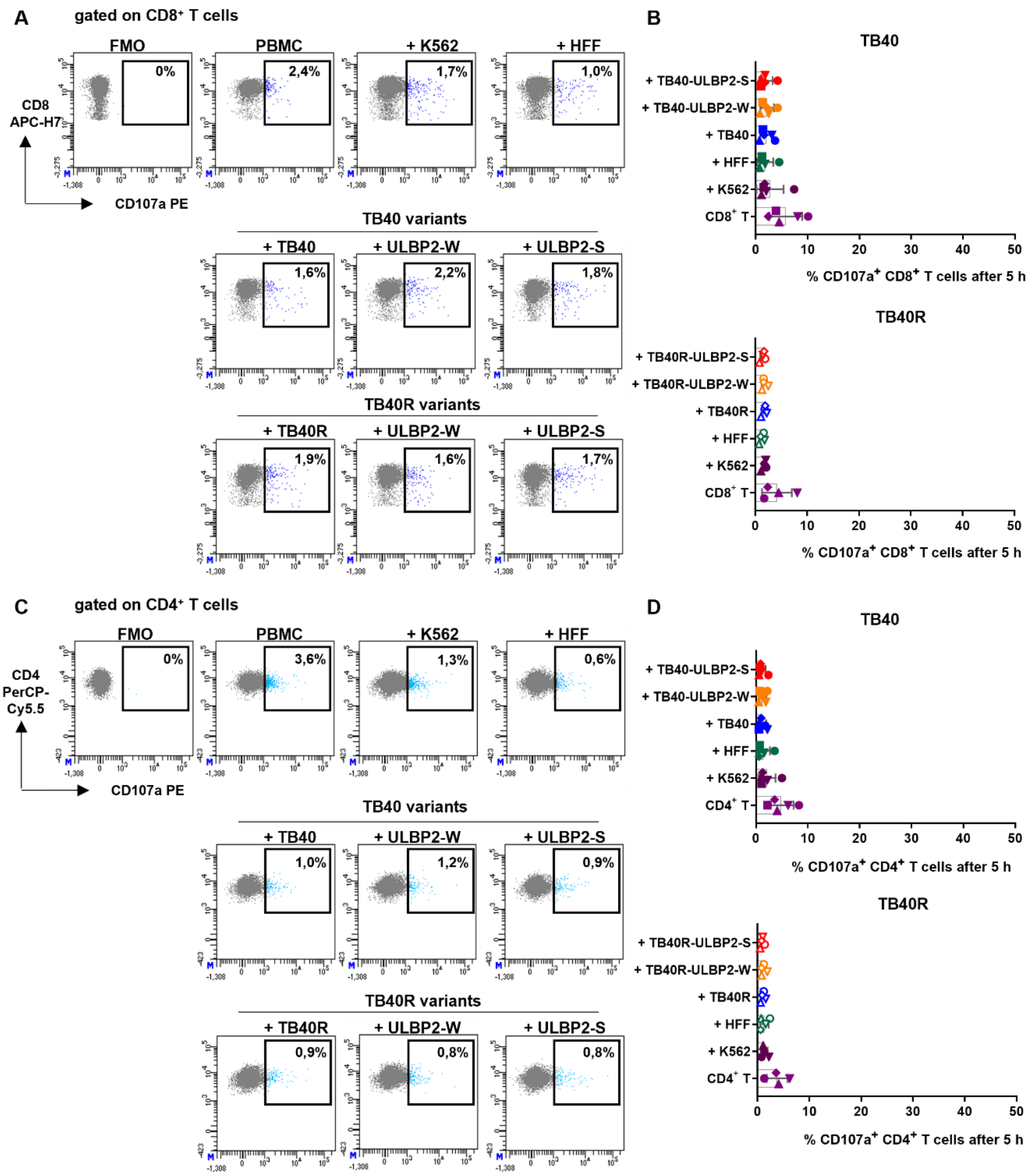

Supplementary Figure 9 Degranulation capacity of CD8$^+$ and CD4$^+$ T cells in response to HCMV-infected cells. PBMC obtained from three healthy HCMV-negative donors were pre-stimulated for 48 h with IL-2 and subsequently added to either uninfected or 5 dpi HCMV-infected HFF for 5 h to analyze the degranulation capacity of T cells (gated by CD3$^+$CD56$^-$, CD4$^+$ or CD8$^+$; representative gating strategy shown in Supplementary Figure 5B). CD107a expression was analyzed by flow cytometry. **(A, C)** Dot plots depicting % of CD107a$^+$CD8$^+$ or CD4$^+$ T cells of one representative donor. **(B, D)** Frequencies of CD107a$^+$CD8$^+$ or CD4$^+$ T cells (mean ± SD, n= 4-5). Each specific symbol represents one donor. The CD107a positive gate was set according to the corresponding NK cell data shown in **Figure 5A-B**.

SUPPLEMENTARY TABLES

Table 1 Primary conjugated antibodies

Target	Fluorochrome	Clone	Company, Cat.-Number
**CD3**	V500	UCHT1	BD Biosciences, 561416
**CD3**	PE-Cy7	UCHT1	Biolegend, 300420
**CD4**	PerCP-Cy5.5	RPA-T4	Biolegend, 300530
**CD8**	PE-Cy7	SK1	BD Biosciences, 335822
**CD8**	APC-H7	SK1	BD Biosciences, 641400
**CD8**	BV650	SK1	Biolegend, 344730
**CD16**	APC-Cy7	3G8	BD Biosciences, 557758
**CD16**	FITC	3G8	Beckman Coulter, B49215
**CD19**	BV421	HIB19	BD Biosciences, 562440/ 562441
**CD25**	FITC	B1.49.9	Beckman Coulter, IM0478U
**CD45**	BV421	HI30	Biolegend, 304032
**CD45**	AF700	HI30	Biolegend, 304024
**CD56**	PE	B159	BD Biosciences, 555516
**CD56**	APC	N901	Beckman Coulter, IM 2474
**CD56**	BV510	NCAM16.2	BD Biosciences, 563041
**CD69**	FITC	FN50	BD Biosciences, 555530
**CD94**	FITC	HP-3D9	BD Biosciences, 555888
**CD96 v2**	AF700	628211	R&D Systems, FAB6119N
**CD107a**	PE	H4A3	BD Biosciences, 555801
**CD107a**	APC	H4A3	BD Biosciences, 560664
**CD158a (KIR2DL/S1)**	FITC	HP-3E4	BD Bioscienes, 340531
**CD158b (KIR2DL/S2/3)**	PE	GL183	Immunotech (B. Coulter), IM2278U
**CD158e (KIR3DL/S1)**	PE	Z27.3.7	Immunotech (B. Coulter), IM3292
**CD159c (NKG2C)**	PE	134591	R&D Systems, FAB138P
**CD159a (NKG2A)**	PE-Cy7	Z199	Beckman Coulter, B10246
**CD226 (DNAM-1)**	PE	DX11	BD Biosciences, 559789
**CD314 (NKG2D)**	APC	1D11	Invitrogen (eBioscience), 17-5878-42
**TIGIT**	BV421	741182	BD Bioscience, 747844
**HLA-DR**	BV421	G46-6	BD Biosciences, 562805
**IgG1**	V500/Amcyan	X40	BD Biosciences, 339185
**IgG1 kappa**	APC eFluor780	P3.6.2.8.1	Invitrogen (eBioscience)
**IgG1**	BV421	MOPC21	Biolegend, 400157
**IgG1**	PE	MOPC21	BD Biosciences, 555749
**IgG1**	FITC	MOPC21	BD Biosciences, 555748
**IgM**	FITC	G20-127	BD Biosciences, 555782
**IgG1**	PE-Cy7	MOPC21	Biolegend, 400126
**IgG1**	PerCP-Cy5.5	P3.6.2.8.1	eBioscience, 45-4714-82
**IgG2a**	APC	X39	BD Biosciences, 340473

Table 2 Primary unconjugated antibodies

Target	Fluorochrome	Clone	Company, Cat.-Number
**Isotype IgG2a mouse**	unconjugated	UPC10	Sigma
**Isotype IgG1 mouse**	unconjugated	MOPC21	Sigma
**Isotype IgG1 rat**	unconjugated	Cad8	Self-production
**ULBP1**	unconjugated	170818	R&D Systems, MAB1380-100
**ULBP-2,-5,-6**	unconjugated	165903	R&D Systems, MAB1298-100
**ULBP3**	unconjugated	166510	R&D Systems, MAB1517-100
**MICA**	unconjugated	159227	R&D Systems, MAB1300-100
**MICB**	unconjugated	236511	R&D Systems, MAB1599-100
**CD155**	unconjugated	D171	Invitrogen, MA5-13493
**CD166**	unconjugated	3A6	BD Bioscience, AB_397209
**CD314 (NKG2D)**	unconjugated	1D11	BD Pharmingen, 552866
**HLA-E**	unconjugated	4D1-2-1	Self-production
**panHLA class I**	unconjugated	W6-32	Self-production

Table 3 Secondary antibodies

Isotype	Fluorochrome	Clone	Company,Cat.-Number
**F(ab')$_2$ Fragment goat anti-mouse IgG + IgM (H+L)**	PE	polyclonal	DIANOVA/Jackson ImmunoResearch, 115-116-068
**F(ab')$_2$ Fragment goat anti-mouse IgG + IgM (H+L)**	APC	polyclonal	DIANOVA/Jackson ImmunoResearch, 115-136-068
**F(ab')$_2$ Fragment goat anti-human IgG, Fc γ Fragment**	APC	polyclonal	DIANOVA/Jackson ImmunoResearch, 109-136-098

Supporting information – References:

(1) Tomić A, Varanasi PR, Golemac M, Malić S, Riese P, Borst EM, Mischak-Weissinger E, Guzmán CA, Krmpotić A, Jonjić S, Messerle M. Activation of Innate and Adaptive Immunity by a Recombinant Human Cytomegalovirus Strain Expressing an NKG2D Ligand. *PLoS Pathog* 2016;12(12):e1006015.

(2) Sinzger C, Hahn G, Digel M, Katona R, Sampaio KL, Messerle M, Hengel H, Koszinowski U, Brune W, Adler B. Cloning and sequencing of a highly productive, endotheliotropic virus strain derived from human cytomegalovirus TB40/E. *J Gen Virol* 2008;89(Pt 2):359–368.

(3) Hammer Q, Rückert T, Borst EM, Dunst J, Haubner A, Durek P, Heinrich F, Gasparoni G, Babic M, Tomic A, Pietra G, Nienen M, Blau IW, Hofmann J, Na I, Prinz I, Koenecke C, Hemmati P, Babel N, Arnold R, Walter J, Thurley K, Mashreghi M, Messerle M, Romagnani C. Peptide-specific recognition of human cytomegalovirus strains controls adaptive natural killer cells. *Nat Immunol* 2018;19(5):453–463.

(4) Tischer BK, Smith GA, Osterrieder N. En passant mutagenesis: a two step markerless red recombination system. *Methods Mol Biol* 2010;634:421–430.

(5) Forte E, Li M, Ayaloglu Butun F, Hu Q, Borst EM, Schipma MJ, Piunti A, Shilatifard A, Terhune SS, Abecassis M, Meier JL, Hummel M. Critical Role for the Human Cytomegalovirus Major Immediate Early Proteins in Recruitment of RNA Polymerase II and H3K27Ac To an Enhancer-Like Element in OriLyt. *Microbiol Spectr* 2023;11(1):e0314422–22.

(6) Elbasani E, Gabaev I, Steinbrück L, Messerle M, Borst EM. Analysis of essential viral gene functions after highly efficient adenofection of cells with cloned human cytomegalovirus genomes. *Viruses* 2014;6(1):354–370.
